# Exploring the relationship between maternal prenatal stress and brain structure in premature neonates

**DOI:** 10.1371/journal.pone.0250413

**Published:** 2021-04-21

**Authors:** Alexandra Lautarescu, Laila Hadaya, Michael C. Craig, Antonis Makropoulos, Dafnis Batalle, Chiara Nosarti, A. David Edwards, Serena J. Counsell, Suresh Victor

**Affiliations:** 1 Department of Perinatal Imaging and Health, Centre for the Developing Brain, School of Biomedical Engineering and Imaging Sciences, King’s College London, London, United Kingdom; 2 Department of Forensic and Neurodevelopmental Sciences, Institute of Psychiatry, Psychology and Neuroscience, King’s College London, London, United Kingdom; 3 Department of Child and Adolescent Psychiatry, Institute of Psychiatry, Psychology and Neuroscience, King’s College London, London, United Kingdom; 4 National Female Hormone Clinic, South London and Maudsley National Health Service Foundation Trust, London, United Kingdom; Western University, CANADA

## Abstract

**Background:**

Exposure to maternal stress in utero is associated with a range of adverse outcomes. We previously observed an association between maternal stress and white matter microstructure in a sample of infants born prematurely. In this study, we aimed to investigate the relationship between maternal trait anxiety, stressful life events and brain volumes.

**Methods:**

221 infants (114 males, 107 females) born prematurely (median gestational age = 30.43 weeks [range 23.57–32.86]) underwent magnetic resonance imaging around term-equivalent age (mean = 42.20 weeks, SD = 1.60). Brain volumes were extracted for the following regions of interest: frontal lobe, temporal lobe, amygdala, hippocampus, thalamus and normalized to total brain volume. Multiple linear regressions were conducted to investigate the relationship between maternal anxiety/stress and brain volumes, controlling for gestational age at birth, postmenstrual age at scan, socioeconomic status, sex, days on total parenteral nutrition. Additional exploratory Tensor Based Morphometry analyses were performed to obtain voxel-wise brain volume changes from Jacobian determinant maps.

**Results and conclusion:**

In this large prospective study, we did not find evidence of a relationship between maternal prenatal stress or trait anxiety and brain volumes. This was the case for both the main analysis using a region-of-interest approach, and for the exploratory analysis using Jacobian determinant maps. We discuss these results in the context of conflicting evidence from previous studies and highlight the need for further research on premature infants, particularly including term-born controls.

## Introduction

Poor maternal mental health during pregnancy represents a global public health problem, affecting 10–35% of pregnant women [1, 2]. Maternal prenatal psychological distress in the form of maternal depression, anxiety, and/or stress has been associated with adverse obstetrical and early behavioural outcomes, and an increased risk of neurodevelopmental and psychiatric disorders [3–7]. The biological basis of these effects is still poorly understood. However, studies by our group and others suggest prenatal maternal stress modulates the neurodevelopment of brain networks that underpin these disorders [8–11].

The brain regions that appear to be most vulnerable to maternal prenatal stress, other forms of early adversity, and psychopathology include the regions of the frontal lobe, temporal lobe, and limbic system [12–17]. These areas are connected by the uncinate fasciculus, and we recently reported an association between maternal stressful life events and increased diffusivity in this tract, in a sample of premature neonates [18].

However, although there is evidence suggesting that maternal prenatal stress affects the development of white matter tracts, evidence for early changes in structural brain development is inconclusive [12]. A small number of studies have examined this relationship in neonates and infants born at term, suggesting no evidence for differences in brain volumes in relation to maternal psychological distress [10, 19, 20]. Several studies have been conducted on older participants (i.e. childhood, adolescence, and adulthood), with the most commonly reported findings being cortical thinning [21–24], and either reductions [25–27], or increases in regional volumes [28–30].

While human studies so far have been inconclusive, animal studies have provided some limited evidence that maternal distress is related to early volume changes in the limbic system, particularly the hippocampus, amygdala, and thalamus [31–36].

We must also consider biological sex as a potential moderator of risk transmission, as several studies have reported volume changes in females, but not males [28, 30, 37]. In utero stress exposure has been associated with higher rates of mood disorders and anxiety [38–40] in females, and behavioural problems [41] and ADHD [6] in males. High maternal cortisol levels at 15 weeks’ gestation has been associated with increased right amygdala volumes and more affective problems in female, but not male, offspring [41].

In summary, although research has reported differences in brain structure in children, adolescents and adults exposed to maternal psychological distress, evidence in infants is inconclusive. To our knowledge, no studies have investigated this relationship in infants born prematurely. Premature birth is associated with changes in brain development [42] and an increased risk of adverse neurodevelopmental and psychiatric outcomes [43, 44]. In order to improve outcomes in these children, it is important to better understand the role that early adverse experiences such as exposure to prenatal stress could have in moderating these associations.

In this study, we investigated the relationship between maternal trait anxiety and stressful life events, and brain volumes in a large sample of infants born prematurely. We have previously shown differences in white matter microstructure in the uncinate fasciculus in this sample [18]. Based on previous literature, we hypothesized that maternal prenatal stress/trait anxiety would be associated with regional volume differences in areas adjacent to the uncinate fasciculus: frontal and temporal lobe volume, amygdala, hippocampus and thalamus. As the direction of effect in the literature is inconsistent (i.e. volumes found to be normal, enlarged, or decreased), we did not hypothesize a direction of effect. Lastly, given the heterogeneity of outcomes associated with maternal stress, as well as the complexity of functional anatomy in the chosen regions of interest (Text in S1 File), we conducted a whole brain analysis using Tensor Based Morphometry.

## Methods and materials

### Participants

Participants were mother-infant dyads who took part of the Evaluation of Preterm Imaging Study (ePRIME, [45]). Ethical approval was obtained from the Hammersmith and Queen Charlotte’s Research Ethics Committee (09/H0707/98) and informed written consent was obtained from all participants. Participants were recruited between April 2010 and July 2013 by screening 3619 admissions to level 1,2 and 3 neonatal units at 14 London Hospitals. Eligibility criteria for the main study included: infant born before 33 weeks gestational age, mother aged over 16 years, not a hospital inpatient, no major congenital malformation, no prior MRI, no care in a centre where preterm MRI was routine, no metallic implants, parents able to speak English, parents not subject to child protection proceedings. The ePrime cohort is representative of the UK NICU population in terms of birth weight, ethnicity, and prevalence of cerebral palsy (6%). Additional information is available in [45].

Data was available for n = 511 infants who were born prematurely (before 33 weeks of gestation) and scanned at term equivalent age. We excluded cases where the postmenstrual age at scan was >45 (n = 48), data was not available for all variables of interest (n = 160), women disclosed alcohol and/or drug abuse during pregnancy (n = 5), or the images showed major focal lesions such as periventricular leukomalacia, haemorrhagic parenchymal infarction, and other ischemic or haemorrhagic lesions (n = 40). In cases where a mother had multiple infants enrolled in the study (i.e. twin and triplet pregnancies), only one infant was randomly included in the final analysis. From the remaining sample, segmentation data for the structures of interest were available for n = 221 (Table 1), and a voxel-wise exploratory analysis using Tensor Based Morphometry was performed on the same 221 participants. The sample partially overlapped (n = 191) with a previous study [18]. Maternal socioeconomic status (SES) values were extracted from the Carstairs index, which takes into consideration variables such as unemployment, car ownership, household overcrowding, and social class [46].

**Table 1:** Obstetric and sociodemographic characteristics (n = 221).

**Maternal Characteristics**	**Reported**	**Values**
Stressful life events score	Median (range)	53 (0–270)
Trait anxiety score	Median (range)	36 (20–67)
Maternal age at scan	Mean (SD)	32.94 (5.70)
Maternal SES	Median (range)	17.44 (1.73–60.58)
Maternal education (years)	N (%)	
16 or less		24 (10.8%)
17–19		30 (13.5%)
19+		156 (70.6%)
Still in full-time education		8 (3.6%)
Not reported		3 (1.3%)
**Infant Characteristics**	**Reported**	**Values**
Infant sex	N (%)	
Male		114 (51.5%)
Female		107 (48.4%)
GA at birth (weeks)	Median (range)	30.43 (23.57–32.86)
PMA at scan (weeks)	Mean (SD)	42.20 (1.60)
Birth weight (grams)	Median (range)	1300 (600–2600)
OFC at birth (cm), n = 192	Median (range)	29.00 (21.80–36)
Number of days on TPN	Median (range)	6 (0–59)
Number of days on ventilation	Median (range)	0 (0–33)

Mean and SD are reported for normally distributed data; median and range are reported for non normally distributed data. GA = gestational age, OFC = Orbitofrontal circumference, PMA = postmenstrual age, SD = standard deviation, SES = socioeconomic status, TPN = Total Parenteral Nutrition, Maternal education = age at leaving formal education. No missing data unless otherwise specified in table.

### Trait anxiety

The State Trait Anxiety Inventory (STAI, [47]) which measures levels of anxiety right now (i.e. state) and in general (i.e. trait), was administered at the time of the MRI scan. The analysis was restricted to trait anxiety, as it measures a relatively stable tendency to be prone to experiencing anxiety and thus extends to the period before birth.

For trait anxiety, missing values were imputed for cases in which a maximum of 10% of questions were missing. We imputed missing values by calculating the average response for the questions that were answered. Missing values were imputed for n = 23 (n = 18 missing 1/20 answers and n = 5 missing 2/20 answers).

### Stressful life events

Stressful life events were assessed using a questionnaire adapted from the Avon Longitudinal Study of Parents and Children [48], which included yes/no answers to a list of potentially stressful life events the participant may have experienced in the year prior to the study visit (e.g. “Arguments with your partner increased”). Events were ranked according to severity [18] based on the Social Readjustment Rating Scale [49] and summed to form a final score that accounts for the number and severity of events experienced (Table J in S1 File). There were no missing data for this variable.

### MR imaging

Magnetic resonance imaging data were acquired using an 8-channel phased array head coil, on a Philips 3T (Philips Medical Systems, Best, The Netherlands) MR system located on the intensive care unit. Imaging data was acquired as follows: Three-dimensional magnetization prepared rapid acquisition gradient echo (repetition time: 17 ms; echo time: 4.6 ms; flip angle: 13°; slice thickness: 0.8 mm; in-plane resolution: 0.82 × 0.82 mm2), T2-weighted turbo spin echo (repetition time: 8670 ms; echo time: 160 ms; flip angle: 90°; slice thickness: 2 mm; in-plane resolution: 0.86 × 0.86 mm2), and single shot echo planar DTI (repetition time: 7536 ms; echo time: 49 ms; flip angle: 90°; slice thickness: 2 mm; in-plane resolution: 2 x 2 mm2, 32 noncollinear gradient directions, b value of 750 s/mm2, 1 non-diffusion-weighted image, b = 0).

An experienced paediatrician supervised all scanning sessions. To enable a successful scan, the majority of infants included in this study were sedated with oral chloral hydrate (25–50 mg/kg) and monitored throughout the scan using pulse oximetry, temperature monitors and electrocardiography. Ear protection was used for all infants, in the form of earplugs molded from a silicone-based putty (President Putty; Coltene Whaledent, Mahwah, NJ) and neonatal earmuffs (MiniMuffs; Natus Medical Inc., San Carlos, CA).

### Segmentations

Images were analysed using an automated processing pipeline optimised for neonates. Following motion correction, bias correction and brain extraction, T2w images were segmented using the Draw-EM algorithm, an open-source software optimised for neonatal brain segmentation [50]. Analysing MR images from infants, and especially preterm infants, poses unique challenges, such as motion, lower contrast-to-noise ratio, and partial volume effects; for a discussion of how these were addressed, see [50].

Based on previous literature and considerations of multiple comparisons issues, the following volumes were chosen as variables of interest: amygdala, hippocampus, thalamus, frontal lobe and temporal lobe (Table A in S1 File). To account for inter-individual differences in brain size, all brain volumes included in the analysis were normalized to total brain volume (i.e. dividing each regional volume by total brain volume).

### Tensor-based morphometry

#### Template construction

A multivariate study-specific template was built using images from a subset of 161 participants. In order to reduce computational load, a smaller subset of 161 images meeting inclusion criteria (i.e. PMA at scan <45 weeks, no major lesions, and of good quality) were used to build the population template for this study. Using the Advanced Normalization Tools (ANTS) software to build the template [51], we applied field bias correction and used the Developing Human Connectome Project 40 weeks’ gestational age T2-weighted [52] and T2 tissue labels templates [50] as the target volumes for the template construction inputs. Iteration limit was set to the default (4 iterations).

#### Registration and log-Jacobian determinants

Images were registered to the study-specific template using the multimodal Symmetric Normalisation (SyN) algorithm from the ANTs software (n = 221) [53]. To improve image registration, two input modalities were used: T2-weighted images and T2-based tissue type segmentation [50]. T2-weighted deformation tensor fields (i.e. warps) from non-linear transformations of the registration process were used to compute a logarithm transformation of Jacobian determinant maps (i.e. deformation tensor field gradients), which reflect volume changes from the template at the voxel-level [54]. Jacobian determinants reflect the degree of transformation (i.e. the expanding or contracting) an image voxel has undergone in order to fit into the template space; therefore, providing information on the relative volumes of brain structures. Smoothing with a 4mm full-width half-maximum Gaussian filter was applied to the log-Jacobian determinants, in order to increase the signal-to-noise ratio. We re-sampled the smoothed log-Jacobian maps from the original isotropic voxel size of 0.5 cm3 to 1 cm3 before running statistical analyses in order to help with computation and memory constraints.

### Statistical analysis

#### Main analysis

Statistical analysis was performed using R [55], with the main packages being psych [56], ggplot2 [57], and hmisc [58]. A minimal dataset and the analysis code including a comprehensive list of packages are available in the (Text in S1 File, S1 Dataset).

We assessed potential covariates using bivariate Spearman’s correlations (Table B in S1 File). Birth weight was excluded as a covariate from the main analysis as it was highly correlated with gestational age (r = .74, p < .001). The number of days on ventilation was also excluded as a covariate in the main analysis as it was highly correlated with the number of days on total parenteral nutrition (r = .60, p = .001), both measures provide information on the health status of the infant, and the distribution of days on total parenteral nutrition was less skewed. Maternal education and number of complications were not correlated with any of the variables of interest and thus were excluded in the main analysis. The regression models used were the same as those used in [18].

Multiple linear regressions were conducted to investigate the relationship between maternal trait anxiety/stress and brain volumes in premature infants. Our models contained the following predictors: stressful life events, trait anxiety, GA, PMA, SES, biological sex, days on total parenteral nutrition. The models were run separately for each dependent variable (frontal lobe grey matter, temporal lobe grey matter, thalamus, amygdala, hippocampus). Correction for multiple comparisons was performed using False Discovery Rate (FDR), and all p values reported below are uncorrected. Unless otherwise specified, all regression models met assumptions for multiple regression (i.e. normality, linearity, homogeneity of variance, uncorrelated predictors, no influential outliers, independence of residuals, [59], Table C in S1 File). One outlier was removed from all regressions due to violating assumptions of normality (days TPN = 59).

#### Exploratory analysis of tensor based morphometry

Voxel-wise statistical analyses were performed using FSL’s randomise nonparametric permutation testing [60]. A general linear model tested for relationships between log-Jacobian values at the voxel level and the outcome variables of interest (maternal prenatal stress and trait anxiety). We included gestational age at birth, postmenstrual age at scan, socioeconomic status, sex and days on total parenteral nutrition as covariates in our model. We ran 10,000 permutations of the data and used 3D Threshold-Free Cluster Enhancement (TFCE) and Family Wise Error (FWE) to correct for multiple comparisons [61]. Voxels with FWE-corrected P-values at a threshold of P<0.05 were considered to be significant.

## Results

### Segmentations

#### Frontal grey matter volume

The model performed better than expected by chance (p < .001) and accounted for 42% of variance in frontal lobe volume (predicted by PMA, with B = .0058 and SES, with B = .00015). There was no association between frontal grey matter volume and either stressful life events (B = .000018, t = 1.27, p = .204) or trait anxiety (B = -.000024, t = -.304, p = .761, Table D in S1 File). An alternative model removing these two variables performed better (R^2^ = .42, AIC = -1338.38) than the original model (R^2^ = .41, AIC = -1336.09), suggesting that the best fit for a model predicting frontal grey matter volume is one without stressful life events or trait anxiety.

**Fig 1:**
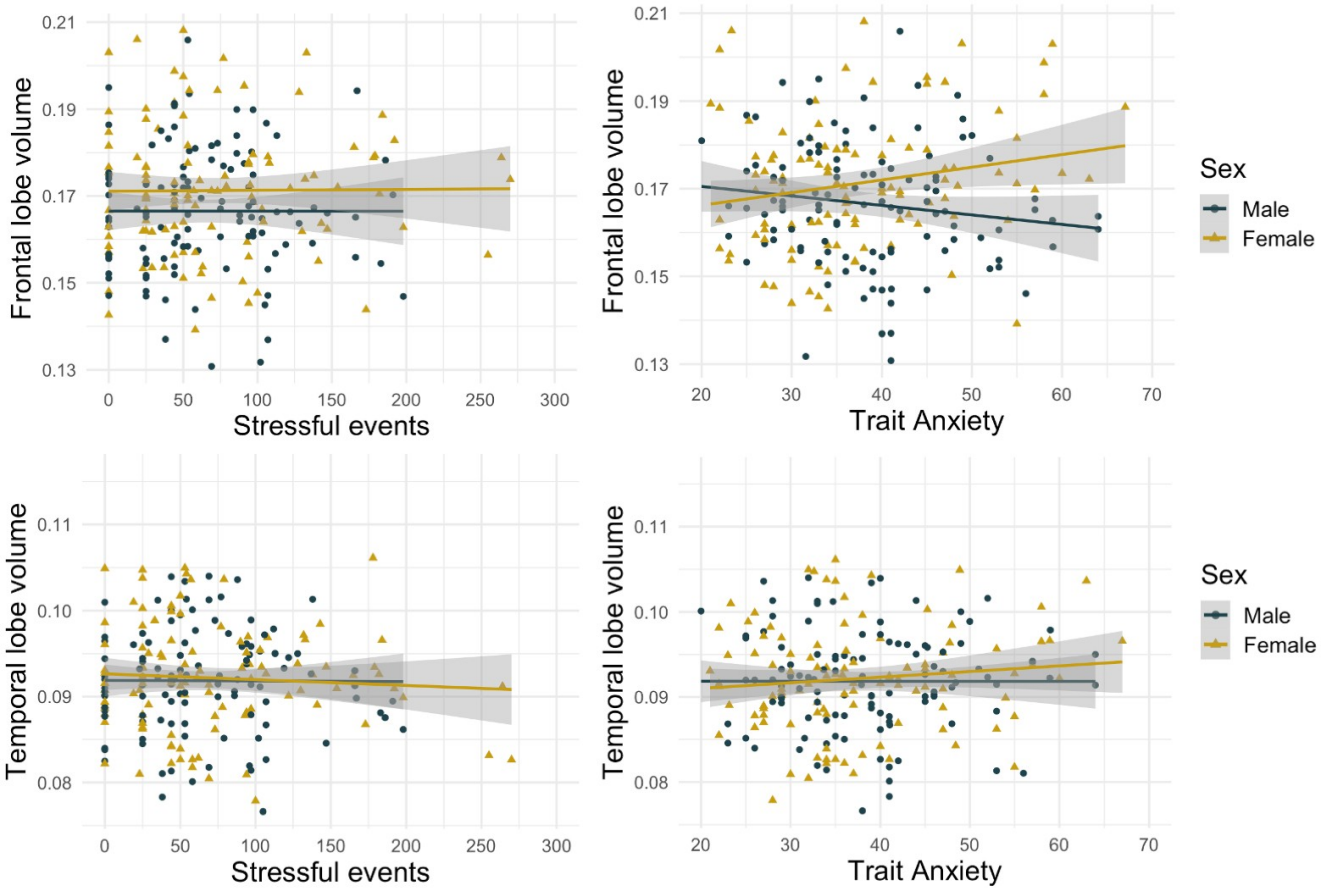
Scatterplots for correlations between maternal trait anxiety/stress and volumes for the frontal and temporal lobes. See Fig A in S1 File for partial regression scatterplots.

Further exploring this relationship with direct Spearman correlations (in the absence of covariates) showed no evidence for a relationship (Fig 1) between frontal grey matter volume and stressful life events (r = .04, p = .593) or trait anxiety (r = .006, p = .929).

#### Temporal grey matter volume

The model did not meet assumptions of homogeneity of variance, and thus we report the heteroscedasticity corrected covariance matrix (Table F in S1 File). The model accounted for 45% of variance in temporal grey matter volume (predicted by PMA, with B = .0025 and SES, with B = .000051). There was no relationship with stressful life events (B = .0000027, t = .495, p = .621) or trait anxiety (B = .0000047, t = .140, p = .889) (Table E in S1 File).

An alternative model removing these two variables performed better (R^2^ = .46, AIC = -.1735.3) than the original model (R^2^ = .46, AIC = -1731.5), suggesting that the best fit for a model predicting temporal grey matter volume is one without stressful life events or trait anxiety.

Further exploring this relationship with direct Spearman correlations (in the absence of covariates) showed no evidence for a relationship (Fig 1) between temporal grey matter volume and stressful life events (r = .04, p = .667) or trait anxiety (r = .05, p = .440).

#### Hippocampal volume

**Fig 2:**
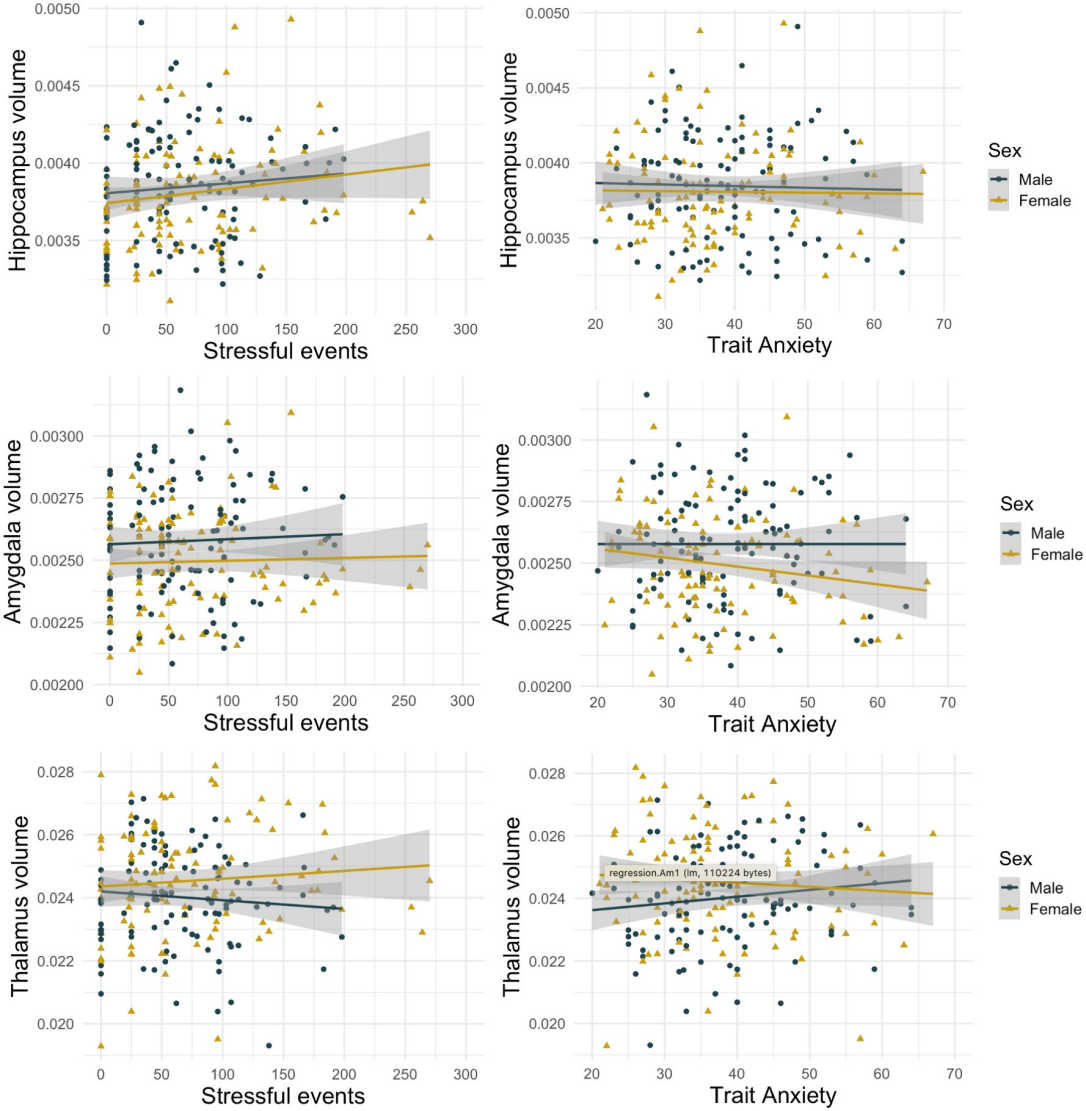
Scatterplots for correlations between maternal trait anxiety/stress and volumes for the hippocampus, amygdala, and thalamus. See Fig B in S1 File for partial regression scatterplots.

Hippocampal volume was not accurately predicted by the model (R^2^ = .06, F(8,211) = 1.58, p = .131), with the only significant predictor being socioeconomic status (B = -.0000040, t = -2.08, p = .039). As the model showed deviations from linearity (Text in S1 File), we repeated the analysis removing 3 outliers (stressful life event scores >250). The new model did not adequately predict hippocampal volume either (R^2^ = .07, F(8,208) = 2.17, p = .031), but stressful life events was a significant predictor (B = .0000012, t = 2.57, p = .011), alongside socioeconomic status (B = -.0000045, t = -2.36, p = .019) (Table G in S1 File). This result did not survive correction for multiple comparisons and visual inspection of the plot suggests no relationship between the variables. An alternative model excluding trait anxiety and stressful life events performed worse (R2 = .05, p = .111), with a higher AIC of -2840.17 compared with -.2842.99. Further exploring this relationship with direct Spearman correlations (in the absence of covariates), suggested a positive correlation between hippocampal volume and stressful life events (r = .16, p = .020), but not trait anxiety (r = -.004, p = .959)(Fig 2).

#### Amygdala volume

For amygdala volume, the model performed better than expected by chance and accounted for 27% of variance in outcome measures (predicted by PMA, with B = -.000064 and SES, with B = -.0000023). There was no relationship with stressful life events (B = -.000000028, t = -.114, p = .909) or trait anxiety (B = -.0000013, t = -1.000, p = .319) (Table H in S1 File). An alternative model removing these two variables performed better (R^2^ = .27, AIC = -3123.95) than the original model (R^2^ = .27, AIC = -3121.05). Direct Spearman correlations showed no evidence for a relationship between amygdala volume and stressful life events (r = .02, p = .770) or trait anxiety (r = -.05, p = .505) (Fig 2). As the model showed deviations from linearity (Text in S1 File), we repeated the analysis removing 3 outliers (stressful life event scores >250). The new model revealed similar results (Table H in S1 File).

#### Thalamus volume

Thalamus volume was not accurately predicted by the model (R^2^ = .08, F(8,210) = 2.40, p = .017). There was no significant relationship between thalamus volume and stressful events (B = -.00000021, t = -.10, p = .920) or trait anxiety (B = -0.00000067, t = -.05, p = .953) (Table I in S1 File). Direct Spearman correlations showed that there was no relationship between thalamus volume and stressful life events (r = -.03, p = .684) or trait anxiety (r = .03, p = .713) (Fig 2).

#### Exploratory analysis subdividing the sample by sex

As visual inspection of scatterplots suggested that the relationship between maternal trait anxiety/stress and brain volumes may be influenced by infant sex, we repeated our analysis subdividing the sample into males and females. There were no significant relationships between maternal trait anxiety/stressful events and infant volume in frontal lobe, temporal lobe, amygdala, thalamus (Text in S1 File).

In our female sample, hippocampal volume was not accurately predicted by the model (R^2^ = .12, F(7,95) = 1.79,p = .097), but the only significant predictor was stressful life events (B = .0000017, t = 2.65, p = .009). This did not survive correction for multiple comparisons. The relationship between hippocampal volume and stressful life events was not observed in males.

### Voxel wise tensor based morphometry results

**Fig 3:**
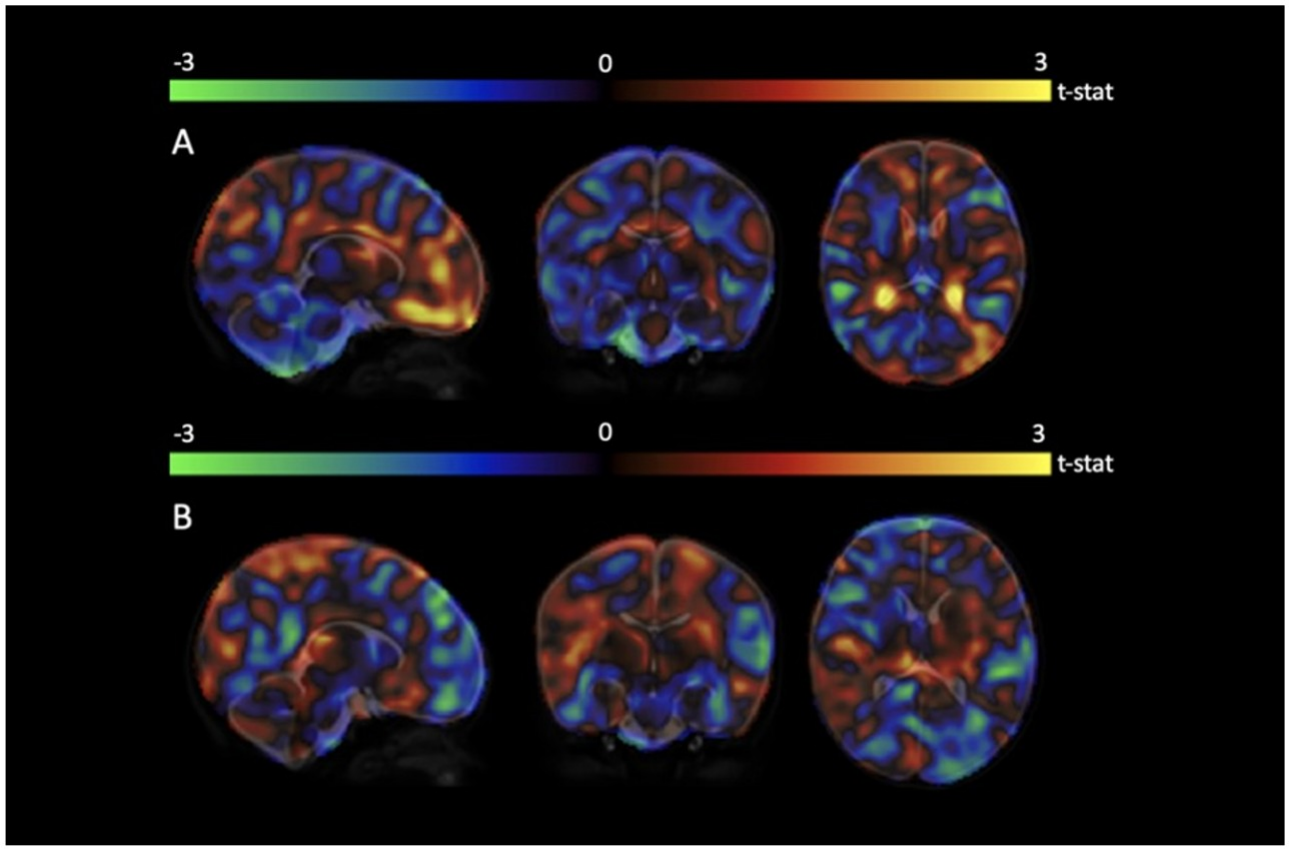
T-statistic maps showing the relationships between voxel-wise log-Jacobian determinants and (a) maternal trait anxiety and (b) stressful life events.

In order to explore whether maternal stress or trait anxiety were associated with neonatal brain volumes at the voxel-level, we conducted Tensor Based Morphometry analyses to obtain Jacobian determinant maps which reflect relative voxel-wise volume changes. Tensor Based Morphometry did not reveal any significant relationships between the smoothed log-Jacobian determinants and maternal prenatal stress or trait anxiety at the FWE p<0.05 threshold. The T-statistic maps (Fig 3) show the test statistic at the voxel level before corrections for multiple comparisons were applied. The whole-brain t-stat maps show generally low t-stat values indicating poor associations between maternal trait anxiety (Fig 3a), or stressful life events (Fig 3b) and log-Jacobian determinants. Nifti files for the t-stat maps are available in the S2 File.

## Discussion

In this study, we did not find evidence for a relationship between maternal stress (i.e. stressful life events and trait anxiety) and grey matter volumes in a large sample of infants born prematurely. These results were consistent across 2 methodologies, using both a whole-brain voxel-wise approach, as well as a region of interest analysis (i.e. hippocampus, amygdala, thalamus, frontal lobe, and temporal lobe).

Interpretation of these findings raises important questions for a field that, to date, has been complicated by inconsistencies between studies along multiple dimensions. These include differences in the samples studied (e.g. age, gender), imaging protocols, definitions of stress, and sample size [12, 62]. Our findings are in line with [10] who reported no difference in right amygdala volume in a large sample of neonates (n = 157) exposed to maternal depression in the second trimester of pregnancy. Similarly, [20] reported no difference in hippocampal volume at birth, but suggested that the hippocampal volume exhibits slower growth in response to exposure to maternal trait anxiety in utero, with smaller volumes being observed at 6 months of age. In a study of exposure to selective serotonin reuptake inhibitors, differences in volume were reported in the right amygdala and right insula [19], but the authors reported no differences in limbic system volumes between untreated depression and controls. Further, [27], in a study of young adults, reported no association between maternal prenatal stress and hippocampal volume, which was instead associated with postnatal anxiety. Studies that have reported associations with maternal distress, primarily regarding cortical thinning in regions of the frontal and temporal lobes [21–24] have been conducted on children rather than infants. Overall, at present, there seems to be no consistent evidence that maternal prenatal stress is associated with neonatal brain volumes, in line with our findings.

This is in stark contrast to the diffusion MRI literature, where studies have consistently reported alterations in limbic and prefrontal microstructure in neonates and infants exposed to maternal psychological distress in utero [9–11, 18, 63]. Further, given that diffusion MRI studies have reported also collecting T2-weighted images, we need to consider whether the lack of studies reporting structural MRI analyses may be driven by the failure to report non-significant findings (i.e. the “file-drawer” problem, [64]). In a recent study published on an overlapping sample [18], we showed differences in white matter microstructure in the uncinate fasciculus in relation to maternal stressful life events. Interestingly, a few of the studies which failed to observe differences in brain structure in relation to maternal psychological distress, reported alterations in white matter microstructure. For example, [10], observed lower fractional anisotropy in the right amygdala of neonates exposed to maternal depression, with no evidence for differences in amygdala volume. Converging evidence suggests that maternal prenatal stress can alter the developing connectome, with differences being most commonly reported in fronto-limbic brain networks (using fMRI and dMRI), with limited evidence for differences in brain structure [62]. Further studies conducted on term-born and preterm infants and reporting on both structural and diffusion MRI are required in order to clarify whether white matter is especially vulnerable to maternal prenatal stress. This is of particular importance given that white matter injury is the most common neuropathology in infants born prematurely [65–67] and white matter may therefore be more vulnerable to additional stressors.

The current study also raised the possibility that the relationship between maternal distress and early brain development may be at least partly influenced by sex differences in the vulnerability to maternal stress in utero. Maternal stressful life events were associated with increased hippocampal volume in the whole sample and in females, but not males; however, these findings were not found to be statistically significant after correction for multiple comparisons.

It is important to highlight that our sample consisted of preterm infants, a population known to have regional brain volume abnormalities [42] and adverse neuropsychiatric and developmental outcomes [44, 68]. We caution against generalizing these findings to infants born at term, and suggest that further studies with term-born controls are needed to further clarify the role that early adverse experiences such as maternal stress may have in moderating the association between preterm birth and adverse outcomes in this vulnerable population.

Although in this study we have examined mean bilateral volumes, several studies of children have reported unilateral differences in volume, such as increased left amygdala volume in girls exposed to pregnancy-specific anxiety, but not boys [37] and greater right amygdala volume in girls exposed to maternal depression, but not boys [30]. Although our analysis was based on mean volumes, the whole-brain analysis did not suggest lateralized differences in volume associated with maternal stress or trait anxiety. Further, other studies that have reported differences in volumes in areas such as the frontal lobe, reported these in very specific areas, such as the mid-dorsolateral frontal cortex [27] or left medial temporal lobe [26]. This may mean that any changes associated with maternal prenatal stress may be more subtle, and thus not affect the overall volume of the frontal or temporal lobes. However, our findings using a voxel-wise whole-brain analysis did not suggest any volume differences associated with maternal stress.

Our findings are not in line with those of [26], who reported decreased amygdala volume, or [28], who reported increased amygdala volume in girls. However, both of these studies were conducted on adult samples, and measures of maternal stress were acquired retrospectively. The biological basis of these potential sex differences is unclear, but may include sex differences in placental functioning, fetal exposure to adrenal hormones and testosterone, as well as various epigenetic mechanisms [69].

Further, there is some evidence to suggest that the child’s development may be more susceptible to maternal pregnancy-specific anxiety, rather than generalized anxiety or stress, as well as that the timing of stress exposure is an important factor to consider [20]. A study [25] suggested that pregnancy anxiety is associated with differences in gray-matter volume at age 6–9, and later reported that neither state anxiety nor depression explained any additional variance in developmental outcomes after accounting for pregnancy-specific anxiety [70]. Future studies should include measures of pregnancy-specific anxiety and assess stress exposure during early, mid, and late gestation.

Although not one of the measures of interest in this study, socio-economic status (which was entered into the regression models as a covariate) was consistently associated with differences in brain volume in our sample of infants born prematurely. Based on these findings, we recommend that future studies should investigate the relationship between socioeconomic status and early brain development, particularly given that low SES is known to be associated with adverse mental health, underreporting of mental health concerns, as well as lack of access to mental health services [71].

It is important to note that although this study was based on subjective self-report measures, the reliability of maternal recall for pregnancy and birth related events appears to be high [72–74], false positive reports of adverse life events are rare [75], and self-reported trait anxiety scores are relatively stable in the perinatal period [76] (See Text in S1 File for further discussion). Future studies should consider including both subjective and laboratory-based measures of stress or anxiety, such as autonomic function, or blood cortisol.

In conclusion, based on our previous findings in an overlapping sample [18], we expected an association between maternal stress and brain volumes in areas adjacent to the uncinate fasciculus tract. To our knowledge, the current study is the first one to examine this relationship in premature infants. In our sample, there is no credible evidence that maternal prenatal stressful life events or trait anxiety influence volumes in the hippocampus, amygdala, thalamus, frontal grey matter or temporal grey matter volume in preterm infants. Our findings are strengthened by an exploratory voxel-wise analysis, and in line with previous literature. Our findings are of particular interest in the context of having reported differences in white matter microstructure in an overlapping sample, using the same statistical methods [18]. It is important to highlight the proximity of our findings to birth, as this minimises the potential confounding influences within the postnatal environment on brain development, which has been a limitation of most prior human studies. We hope that these findings can contribute to a more balanced view of the literature and inform further research into maternal stress and early brain development.

## Supporting information

S1 File (DOCX)

S2 File T-stat maps. (ZIP)

S1 Dataset De-identified research dataset. (CSV)

## References

[1] Almond P. Postnatal depression: a global public health perspective. Perspectives in public health. 2009 9;129(5):221–7. 10.1177/1757913909343882 19788165

[2] Rubin LP. Maternal and pediatric health and disease: integrating biopsychosocial models and epigenetics. Pediatric research. 2016 1;79(1):127–35. 10.1038/pr.2015.203 26484619

[3] Gentile S. Untreated depression during pregnancy: Short-and long-term effects in offspring. A systematic review. Neuroscience. 2017 2 7;342:154–66. 10.1016/j.neuroscience.2015.09.001 26343292

[4] Manzari N, Matvienko-Sikar K, Baldoni F, O’Keeffe GW, Khashan AS. Prenatal maternal stress and risk of neurodevelopmental disorders in the offspring: a systematic review and meta-analysis. Social psychiatry and psychiatric epidemiology. 2019 7 20:1–1. 10.1007/s00127-019-01745-3 31324962

[5] Stein A, Pearson RM, Goodman SH, Rapa E, Rahman A, McCallum M et al. Effects of perinatal mental disorders on the fetus and child. The Lancet. 2014 11 15;384(9956):1800–19. 10.1016/S0140-6736(14)61277-0 25455250

[6] Van den Bergh BR, van den Heuvel MI, Lahti M, Braeken M, de Rooij SR, Entringer S et al. Prenatal developmental origins of behavior and mental health: The influence of maternal stress in pregnancy. Neuroscience & Biobehavioral Reviews. 2017 7 28. 10.1016/j.neubiorev.2017.07.003 28757456

[7] Waters CS, Hay DF, Simmonds JR, van Goozen SH. Antenatal depression and children’s developmental outcomes: potential mechanisms and treatment options. European child & adolescent psychiatry. 2014 10 1;23(10):957–71. 10.1007/s00787-014-0582-3 25037152

[8] Dean DC, Planalp EM, Wooten W, Kecskemeti SR, Adluru N, Schmidt CK et al. Association of prenatal maternal depression and anxiety symptoms with infant white matter microstructure. JAMA pediatrics. 2018 10 1;172(10):973–81. 10.1001/jamapediatrics.2018.2132 30177999 PMC6190835

[9] Posner J, Cha J, Roy AK, Peterson BS, Bansal R, Gustafsson HC et al. Alterations in amygdala–prefrontal circuits in infants exposed to prenatal maternal depression. Translational psychiatry. 2016 11;6(11):e935. 10.1038/tp.2016.146 27801896 PMC5314110

[10] Rifkin-Graboi A, Bai J, Chen H, Hameed WB, Sim LW, Tint MT et al. Prenatal maternal depression associates with microstructure of right amygdala in neonates at birth. Biological psychiatry. 2013 12 1;74(11):837–44. 10.1016/j.biopsych.2013.06.019 23968960

[11] Rifkin-Graboi A, Meaney MJ, Chen H, Bai J, Hameed WB, Tint MT et al. Antenatal maternal anxiety predicts variations in neural structures implicated in anxiety disorders in newborns. Journal of the American Academy of Child & Adolescent Psychiatry. 2015 4 1;54(4):313–21. 10.1016/j.jaac.2015.01.013 25791148

[12] Lautarescu A, Craig MC, Glover V. Prenatal stress: Effects on fetal and child brain development. In International Review of Neurobiology 2020 1 1 (Vol. 150, pp. 17–40). Academic Press. 10.1016/bs.irn.2019.11.002 32204831

[13] Duan C, Hare MM, Staring M, Deligiannidis KM. Examining the relationship between perinatal depression and neurodevelopment in infants and children through structural and functional neuroimaging research. International Review of Psychiatry. 2019 4 3;31(3):264–79. 10.1080/09540261.2018.1527759 30701993 PMC6594877

[14] Zhang FF, Peng W, Sweeney JA, Jia ZY, Gong QY. Brain structure alterations in depression: psychoradiological evidence. CNS neuroscience & therapeutics. 2018 11;24(11):994–1003. 10.1111/cns.12835 29508560 PMC6489983

[15] Bick J, Nelson CA. Early adverse experiences and the developing brain. Neuropsychopharmacology. 2016 1;41(1):177–96. 10.1038/npp.2015.252 26334107 PMC4677140

[16] Teicher MH, Samson JA, Anderson CM, Ohashi K. The effects of childhood maltreatment on brain structure, function and connectivity. Nature Reviews Neuroscience. 2016 10;17(10):652. 10.1038/nrn.2016.111 27640984

[17] Duval ER, Javanbakht A, Liberzon I. Neural circuits in anxiety and stress disorders: a focused review. Therapeutics and clinical risk management. 2015;11:115. 10.2147/TCRM.S48528 25670901 PMC4315464

[18] Lautarescu A, Pecheva D, Nosarti C, Nihouarn J, Zhang H, Victor S et al. Maternal prenatal stress is associated with altered uncinate fasciculus microstructure in premature neonates. Biological psychiatry. 2020 3 15;87(6):559–69. 10.1016/j.biopsych.2019.08.010 31604519 PMC7016501

[19] Lugo-Candelas C, Cha J, Hong S, Bastidas V, Weissman M, Fifer WP et al. Associations between brain structure and connectivity in infants and exposure to selective serotonin reuptake inhibitors during pregnancy. JAMA pediatrics. 2018 6 1;172(6):525–33. 10.1001/jamapediatrics.2017.5227 29630692 PMC6137537

[20] Qiu A, Rifkin-Graboi A, Chen H, Chong YS, Kwek K, Gluckman PD et al. Maternal anxiety and infants’ hippocampal development: timing matters. Translational psychiatry. 2013 9;3(9):e306. 10.1038/tp.2013.79 24064710 PMC3784768

[21] Lebel C, Walton M, Letourneau N, Giesbrecht GF, Kaplan BJ, Dewey D. Prepartum and postpartum maternal depressive symptoms are related to children’s brain structure in preschool. Biological psychiatry. 2016 12 1;80(11):859–68. 10.1016/j.biopsych.2015.12.004 26822800

[22] Davis EP, Hankin BL, Glynn LM, Head K, Kim DJ, Sandman CA. Prenatal maternal stress, child cortical thickness, and adolescent depressive symptoms. Child development. 2020 3;91(2):e432–50. 10.1111/cdev.13252 31073997

[23] El Marroun H, Tiemeier H, Muetzel RL, Thijssen S, van der Knaap NJ, Jaddoe VW et al. Prenatal exposure to maternal and paternal depressive symptoms and brain morphology: A population‐based prospective neuroimaging study in young children. Depression and anxiety. 2016 7;33(7):658–66. 10.1002/da.22524 27163186

[24] Sandman CA, Buss C, Head K, Davis EP. Fetal exposure to maternal depressive symptoms is associated with cortical thickness in late childhood. Biological psychiatry. 2015 2 15;77(4):324–34. 10.1016/j.biopsych.2014.06.025 25129235 PMC4289467

[25] Buss C, Davis EP, Muftuler LT, Head K, Sandman CA. High pregnancy anxiety during mid-gestation is associated with decreased gray matter density in 6–9-year-old children. Psychoneuroendocrinology. 2010 1 1;35(1):141–53. 10.1016/j.psyneuen.2009.07.010 19674845 PMC2795128

[26] Favaro A, Tenconi E, Degortes D, Manara R, Santonastaso P. Neural correlates of prenatal stress in young women. Psychological medicine. 2015 9 1;45(12):2533. 10.1017/S003329171500046X 25786412

[27] Marečková K, Klasnja A, Bencurova P, Andrýsková L, Brázdil M, Paus T. Prenatal stress, mood, and gray matter volume in young adulthood. Cerebral Cortex. 2019 3 1;29(3):1244–50. 10.1093/cercor/bhy030 29425268 PMC6373666

[28] Jones SL, Dufoix R, Laplante DP, Elgbeili G, Patel R, Chakravarty MM et al. Larger amygdala volume mediatesx the association between prenatal maternal stress and higher levels of externalizing behaviors: sex specific effects in project ice storm. Frontiers in human neuroscience. 2019 5 14;13:144. 10.3389/fnhum.2019.00144 31156408 PMC6528106

[29] McQuaid GA, Darcey VL, Avalos MF, Fishbein DH, VanMeter JW. Altered cortical development and psychiatric symptom risk in adolescents exposed to maternal stress in utero. bioRxiv. 2019 1 1:540930. 10.1016/j.bbr.2019.112145 PMC10561894 31400378

[30] Wen DJ, Poh JS, Ni SN, Chong YS, Chen H, Kwek K et al. Influences of prenatal and postnatal maternal depression on amygdala volume and microstructure in young children. Translational psychiatry. 2017 4;7(4):e1103-. 10.1038/tp.2017.74 28440816 PMC5416711

[31] Coe CL, Kramer M, Czéh B, Gould E, Reeves AJ, Kirschbaum C, et al. Prenatal stress diminishes neurogenesis in the dentate gyrus of juvenile rhesus monkeys. Biological psychiatry. 2003 11 15;54(10):1025–34. 10.1016/s0006-3223(03)00698-x 14625144

[32] Kraszpulski M, Dickerson PA, Salm AK. Prenatal stress affects the developmental trajectory of the rat amygdala. Stress. 2006 1 1;9(2):85–95. 10.1080/10253890600798109 16895832

[33] Szuran T, Zimmermann E, Welzl H. Water maze performance and hippocampal weight of prenatally stressed rats. Behavioural brain research. 1994 12 15;65(2):153–5. 10.1016/0166-4328(94)90100-7 7718147

[34] Tamura M, Sajo M, Kakita A, Matsuki N, Koyama R. Prenatal stress inhibits neuronal maturation through downregulation of mineralocorticoid receptors. Journal of Neuroscience. 2011 8 10;31(32):11505–14. 10.1523/JNEUROSCI.3447-10.2011 21832180 PMC6623141

[35] Uno H, Tarara R, Else JG, Suleman MA, Sapolsky RM. Hippocampal damage associated with prolonged and fatal stress in primates. Journal of Neuroscience. 1989 5 1;9(5):1705–11. 10.1523/JNEUROSCI.09-05-01705.1989 2723746 PMC6569823

[36] Yoshii T, Oishi N, Ikoma K, Nishimura I, Sakai Y, Matsuda K et al. Brain atrophy in the visual cortex and thalamus induced by severe stress in animal model. Scientific reports. 2017 10 6;7(1):1–2. 28986553 10.1038/s41598-017-12917-z PMC5630603

[37] Acosta H, Tuulari JJ, Scheinin NM, Hashempour N, Rajasilta O, Lavonius TI et al. Maternal pregnancy-related anxiety is associated with sexually dimorphic alterations in amygdala volume in four-year-old children. Frontiers in behavioral neuroscience. 2019;13:175. 10.3389/fnbeh.2019.00175 31447658 PMC6691065

[38] Baker LM, Williams LM, Korgaonkar MS, Cohen RA, Heaps JM, Paul RH. Impact of early vs. late childhood early life stress on brain morphometrics. Brain imaging and behavior. 2013 6 1;7(2):196–203. 10.1007/s11682-012-9215-y 23247614 PMC8754232

[39] MacMillan HL, Fleming JE, Streiner DL, Lin E, Boyle MH, Jamieson E et al. Childhood abuse and lifetime psychopathology in a community sample. American Journal of Psychiatry. 2001 11 1;158(11):1878–83. 10.1176/appi.ajp.158.11.1878 11691695

[40] Pitzer M, Jennen-Steinmetz C, Esser G, Schmidt MH, Laucht M. Prediction of preadolescent depressive symptoms from child temperament, maternal distress, and gender: results of a prospective, longitudinal study. Journal of Developmental & Behavioral Pediatrics. 2011 1 1;32(1):18–26. 20829711 10.1097/DBP.0b013e3181f4a474

[41] Gerardin P, Wendland J, Bodeau N, Galin A, Bialobos S, Tordjman S et al. Depression during pregnancy: is the developmental impact earlier in boys? A prospective case-control study. The Journal of clinical psychiatry. 2010 11 30;72(3):378–87. 21208585 10.4088/JCP.09m05724blu

[42] Peterson BS, Vohr B, Staib LH, Cannistraci CJ, Dolberg A, Schneider KC et al. Regional brain volume abnormalities and long-term cognitive outcome in preterm infants. Jama. 2000 10 18;284(15):1939–47. 10.1001/jama.284.15.1939 11035890

[43] Johnson S, Marlow N. Preterm birth and childhood psychiatric disorders. Pediatric research. 2011 5;69(8):11–8. 10.1203/PDR.0b013e318212faa0 21289534

[44] Nosarti C, Reichenberg A, Murray RM, Cnattingius S, Lambe MP, Yin L et al. Preterm birth and psychiatric disorders in young adult life. Archives of general psychiatry. 2012 6 1;69(6):610–7. 10.1001/archgenpsychiatry.2011.1374 22660967

[45] Edwards AD, Redshaw ME, Kennea N, Rivero-Arias O, Gonzales-Cinca N, Nongena P et al. Effect of MRI on preterm infants and their families: a randomised trial with nested diagnostic and economic evaluation. Archives of Disease in Childhood-Fetal and Neonatal Edition. 2018 1 1;103(1):F15–21. 28988160 10.1136/archdischild-2017-313102 PMC5750369

[46] Carstairs V, Morris R. *Deprivation and Health in Scotland*. 1991. Aberdeen University Press 2394583

[47] Spielberger CD. Manual for the State-Trait Anxiety Inventory STAI (form Y)(" self-evaluation questionnaire").

[48] Golding J, Pembrey M, Jones R. ALSPAC—the Avon Longitudinal Study of Parents and Children. I. Study methodology. Paediatric and perinatal epidemiology. 2001 1 1;15(1):74–87. 10.1046/j.1365-3016.2001.00325.x 11237119

[49] Holmes TH, Rahe RH. The social readjustment rating scale. Journal of psychosomatic research. 1967. 10.1016/0022-3999(67)90010-4 6059863

[50] Makropoulos A, Gousias IS, Ledig C, Aljabar P, Serag A, Hajnal JV et al. Automatic whole brain MRI segmentation of the developing neonatal brain. IEEE transactions on medical imaging. 2014 5 6;33(9):1818–31. 10.1109/TMI.2014.2322280 24816548

[51] Avants BB, Tustison NJ, Song G, Cook PA, Klein A, Gee JC. A reproducible evaluation of ANTs similarity metric performance in brain image registration. Neuroimage. 2011 2 1;54(3):2033–44. 10.1016/j.neuroimage.2010.09.025 20851191 PMC3065962

[52] Schuh A, Makropoulos A, Robinson EC, Cordero-Grande L, Hughes E, Hutter J et al. Unbiased construction of a temporally consistent morphological atlas of neonatal brain development. bioRxiv. 2018 1 1:251512.

[53] Avants BB, Epstein CL, Grossman M, Gee JC. Symmetric diffeomorphic image registration with cross-correlation: evaluating automated labeling of elderly and neurodegenerative brain. Medical image analysis. 2008 2 1;12(1):26–41. 10.1016/j.media.2007.06.004 17659998 PMC2276735

[54] Avants B, Gee JC. Geodesic estimation for large deformation anatomical shape averaging and interpolation. Neuroimage. 2004 1 1;23:S139–50. 10.1016/j.neuroimage.2004.07.010 15501083

[55] Team R Core. R: A language and environment for statistical computing.

[56] Revelle WR. psych: Procedures for personality and psychological research.

[57] Wickham H. ggplot2: elegant graphics for data analysis. springer; 2016 6 8.

[58] Harrell FE Jr, Harrell MF Jr. Package ‘Hmisc’. CRAN2018. 2019 1 25;2019:235–6.

[59] Navarro D. Learning statistics with r: A tutorial for psychology students and other beginners: version 0.5. Adelaide, Australia: University of Adelaide; 2013.

[60] Winkler AM, Ridgway GR, Webster MA, Smith SM, Nichols TE. Permutation inference for the general linear model. Neuroimage. 2014 5 15;92:381–97. 10.1016/j.neuroimage.2014.01.060 24530839 PMC4010955

[61] Smith SM, Nichols TE. Threshold-free cluster enhancement: addressing problems of smoothing, threshold dependence and localisation in cluster inference. Neuroimage. 2009 1 1;44(1):83–98. 10.1016/j.neuroimage.2008.03.061 18501637

[62] Scheinost D, Sinha R, Cross SN, Kwon SH, Sze G, Constable RT, et al. Does prenatal stress alter the developing connectome?. Pediatric research. 2017 1;81(1–2):214–26. 10.1038/pr.2016.197 27673421 PMC5313513

[63] Dennis EL, Singh A, Corbin CK, Jahanshad N, Ho TC, King LS et al. Associations between maternal depression and infant fronto-limbic connectivity. In2019 IEEE 16th International Symposium on Biomedical Imaging (ISBI 2019) 2019 Apr 8 (pp. 126–130). IEEE.

[64] Jennings RG, Van Horn JD. Publication bias in neuroimaging research: implications for meta-analyses. Neuroinformatics. 2012 1 1;10(1):67–80. 10.1007/s12021-011-9125-y 21643733 PMC4368431

[65] Anjari M, Srinivasan L, Allsop JM, Hajnal JV, Rutherford MA, Edwards AD et al. Diffusion tensor imaging with tract-based spatial statistics reveals local white matter abnormalities in preterm infants. Neuroimage. 2007 4 15;35(3):1021–7. 10.1016/j.neuroimage.2007.01.035 17344066

[66] Barnett ML, Tusor N, Ball G, Chew A, Falconer S, Aljabar P et al. Exploring the multiple-hit hypothesis of preterm white matter damage using diffusion MRI. NeuroImage: Clinical. 2018 1 1;17:596–606. 10.1016/j.nicl.2017.11.017 29234596 PMC5716951

[67] Ment LR, Hirtz D, Hüppi PS. Imaging biomarkers of outcome in the developing preterm brain. The Lancet Neurology. 2009 11 1;8(11):1042–55. 10.1016/S1474-4422(09)70257-1 19800293

[68] Allen MC. Neurodevelopmental outcomes of preterm infants. *Current opinion in neurology*. 2008; 21(2), 123–128. 10.1097/WCO.0b013e3282f88bb4 18317268

[69] Weinstock M. The long-term behavioural consequences of prenatal stress. Neuroscience & Biobehavioral Reviews. 2008 8 1;32(6):1073–86. 10.1016/j.neubiorev.2008.03.002 18423592

[70] Buss C, Davis EP, Hobel CJ, Sandman CA. Maternal pregnancy-specific anxiety is associated with child executive function at 6–9 years age. Stress. 2011 11 1;14(6):665–76. 10.3109/10253890.2011.623250 21995526 PMC3222921

[71] Goyal D, Gay C, Lee KA. How much does low socioeconomic status increase the risk of prenatal and postpartum depressive symptoms in first-time mothers?. Women’s Health Issues. 2010 3 1;20(2):96–104. 10.1016/j.whi.2009.11.003 20133153 PMC2835803

[72] Sou SC, Chen WJ, Hsieh WS, Jeng SF. Severe obstetric complications and birth characteristics in preterm or term delivery were accurately recalled by mothers. Journal of clinical epidemiology. 2006 4 1;59(4):429–35. 10.1016/j.jclinepi.2005.08.010 16549266

[73] Tomeo CA, Rich-Edwards JW, Michels KB, Berkey CS, Hunter DJ, Frazier AL et al. Reproducibility and validity of maternal recall of pregnancy-related events. Epidemiology. 1999 11 1:774–7. 10535796

[74] Quigley MA, Hockley C, Davidson LL. Agreement between hospital records and maternal recall of mode of delivery: evidence from 12 391 deliveries in the UK Millennium Cohort Study. BJOG: An International Journal of Obstetrics & Gynaecology. 2007 2;114(2):195–200. 10.1111/j.1471-0528.2006.01203.x 17166217

[75] Hardt J, Rutter M. Validity of adult retrospective reports of adverse childhood experiences: review of the evidence. Journal of child psychology and psychiatry. 2004 2;45(2):260–73. 10.1111/j.1469-7610.2004.00218.x 14982240

[76] Grant KA, McMahon C, Austin MP. Maternal anxiety during the transition to parenthood: a prospective study. Journal of affective disorders. 2008 5 1;108(1–2):101–11. 10.1016/j.jad.2007.10.002 18001841

