# Supplementary material for: Exploring the relationship between maternal prenatal stress and brain structure in premature neonates
Source: PLoS One. 2021 Apr 21;16(4):e0250413. doi: 10.1371/journal.pone.0250413 (PMC8059832; doi:10.1371/journal.pone.0250413)
Supplement: S1 File — (DOCX) [file pone.0250413.s001.docx]

**S1 File**

**Additional information regarding data and code**

The full analysis code is available at https://osf.io/4yzpr/.

As our data contained sensitive and potentially identifiable information, we were unable to share the full anonymised dataset necessary to replicate the study findings. We were able to share a dataset (S1 Dataset) containing the following variables:

- EventSum – The total score for the stressful life events measure
- STAI-TRSum – Total score for the trait anxiety measure (STAI-TR)
- GA – Gestational age at birth
- PMA – Postmenstrual age at scan
- IMD_SES – Socioeconomic status
- SEX – infant sex (1=male, 2=female)
- DAYS_TPN – Days on total parenteral nutrition
- FrontalGMbyTBV – Frontal lobe volume relative to total brain volume
- TempGMbyTBV – Temporal lobe volume relative to total brain volume
- ThalamusbyTBV – Thalamus volume relative to total brain volume
- AmygdalabyTBV – Amygdala volume relative to total brain volume
- HippocampusByTBV – Hippocampal volume relative to total brain volume

The variables above represent the vast majority of variables included in the regression models reported in this manuscript. We were unable to share the variable “maternal age”, as this combination of variables would increase the risk of the data being identifiable. We have conducted a sensitivity analysis removing “maternal age” from the regression models, with no major differences in the results (i.e. there was still no significant relationship between maternal stress or anxiety and any of the volumes of interest).

**Additional figures**

Figure A. Partial regression scatterplots showing the relationship between stressful life events (left) or trait anxiety (right) and volumes for the frontal and temporal lobe, while holding the other predictors constant (i.e., gestational age at birth, postmenstrual age at scan, socioeconomic status, total parenteral nutrition, maternal age, sex). Points on the scatterplot represent residuals and the regression line includes standard error bars.


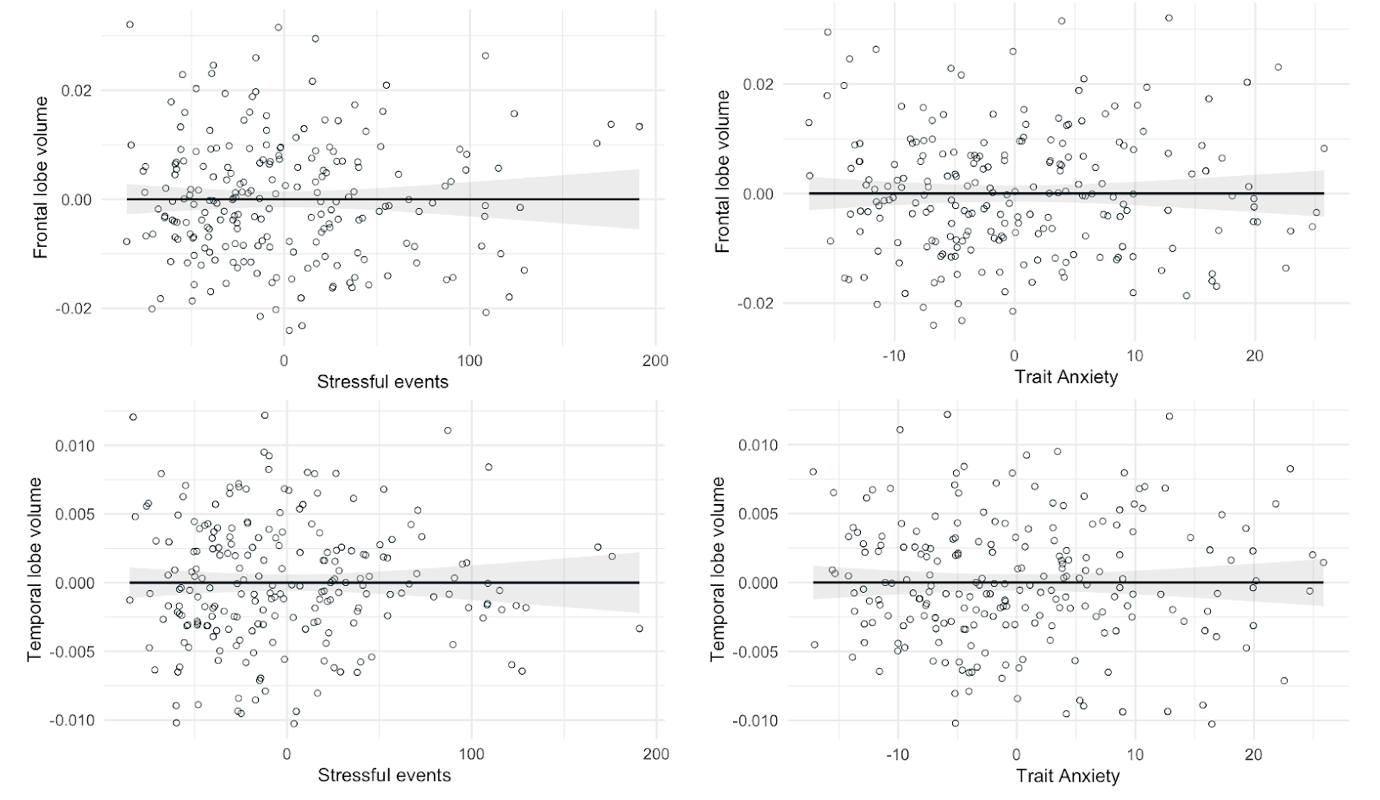


Figure B. Partial regression scatterplots showing the relationship between stressful life events (left) or trait anxiety (right) and volumes for the hippocampus, amygdala, and thalamus, while holding the other predictors constant (i.e., gestational age at birth, postmenstrual age at scan, socioeconomic status, total parenteral nutrition, maternal age, sex). Points on the scatterplot represent residuals and the regression line includes standard error bars.


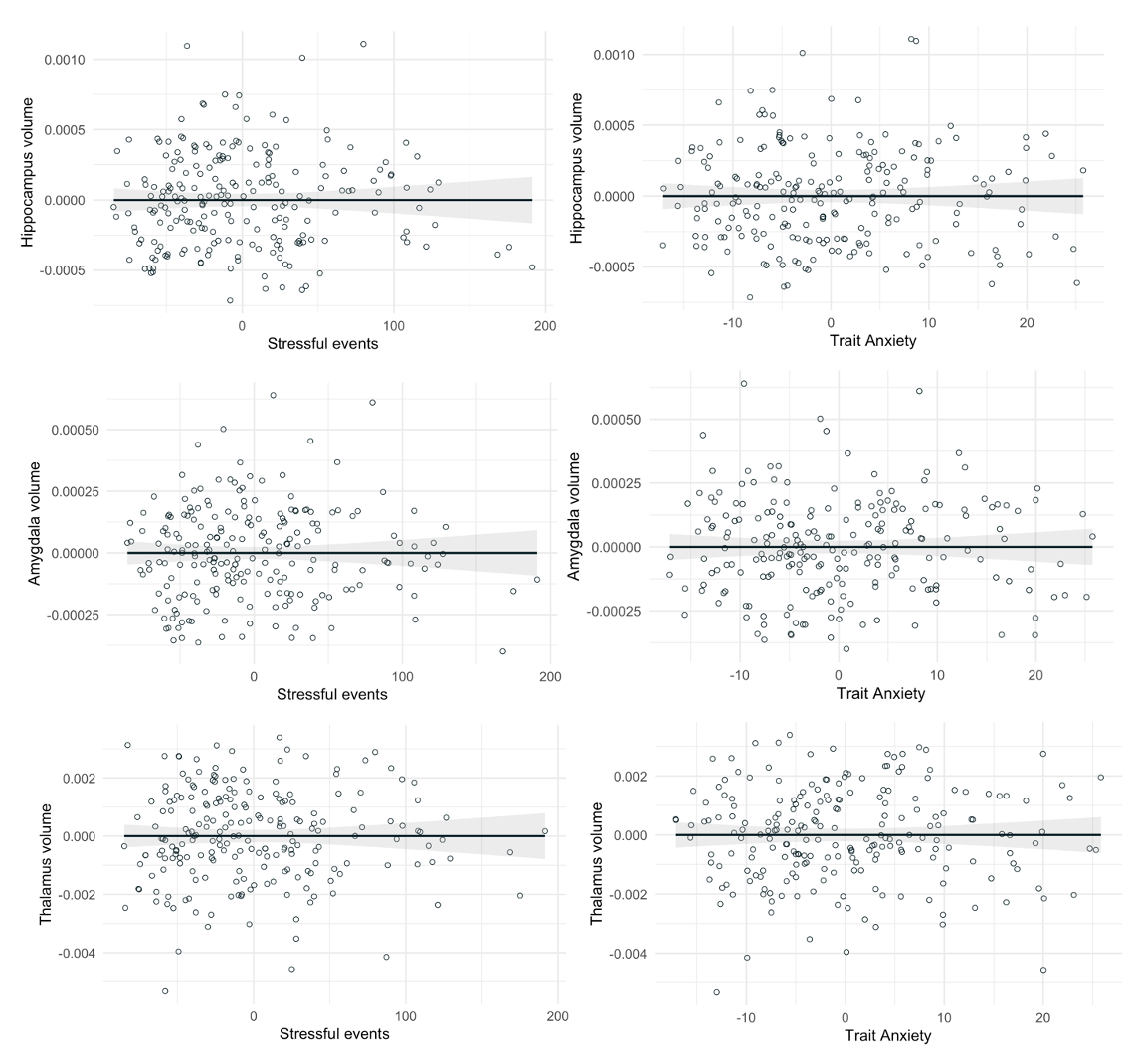


**Additional descriptives**

**Table A. Median and range for brain volumes**

| Volumes | Median | Range |
| --- | --- | --- |
| TBV | 374190.88 | 271016.66 – 487652.31 |
| Frontal lobe – absolute  -relative | 63195.62  0.168 | 35709.18 – 95088.9  0.130 - 0.208 |
| Temporal lobe-absolute  -relative | 34791.19  0.092 | 22571.12- 47395.79  0.076- 0.116 |
| Thalamus-absolute  -relative | 9055.66  0.024 | 6109.08-11761.02  0.016-0.028 |
| Amygdala-absolute  -relative | 943.73  0.002 | 696.61-1225.21  0.002-0.003 |
| Hippocampus-absolute  -relative | 1423.73  0.003 | 916.48-1940.84  0.003-0.004 |

**Table B. Relationship between potential covariates and brain volumes**

|  | TBV | Frontal lobe | Temporal lobe | Thalamus | Amygdala | Hippocampus |
| --- | --- | --- | --- | --- | --- | --- |
| GA at birth | r=.14  p=.043* | r=-.12  p=.069 | r=-.23  p=<.001* | r=-.16  p=.016* | r=.10  p=.126 | r=-.09  p=.171 |
| PMA at scan | r=.42  p=<.001* | r=.63  p=<.001* | r=.65  p=<.001* | r=.08  p=.261 | r=-.50  <.001* | r=-.09  p=.169 |
| Birth weight | r=.36  p<.001* | r=-.12  p=.086 | r=-.18  p=.006* | r=-.34  p=<.001* | r=-.01  p=.932 | r=-.11  p=.110 |
| SES | r=-.05  p=.471 | r=.14  p=.035* | r=.07  p=.298 | r=-.02  p=.818 | r=-.14  .034* | r=-.14  p=.039* |
| Maternal age | r=.21  p=.001* | r=.01  p=.894 | r=.05  p=.489 | r=-.02  p=.741 | r=-.10  p=.138 | r=-.04  p=.530 |
| Maternal education | r=.11  p=.095 | r=-.10  p=.126 | r=-.05  p=.455 | r=-.07  p=.276 | r=.07  p=.312 | r=-.07  p=.294 |
| Complications | r=-.01  p=.920 | r=.08  p=.237 | r=.04  p=.518 | r=.09  p=.174 | r=-.04  p=.508 | r=-.03  p=.613 |
| Duration of TPN (days) | r=-.31  p<.001* | r=.11  p=.093 | r=.18  p=.008* | r=.18  p=.005* | r=-.07  p=.271 | r=.07  p=.298 |
| Duration of ventilation (days) | r=-.20  p=.003* | r=.16  p=.020* | r=.29  p=.001* | r=.14  p=.034* | r=-.08  p=.217 | r=.14  p=.034* |

SES= Socioeconomic status, TBV=total brain volume. With the exception of TBV, all volumes are relative.

**Table C. Assumptions for multiple regression**

| **Issue** | **Tested** | **Frontal** | **Temporal** | **Amygdala** | **Hippocampus** | **Thalamus** |
| --- | --- | --- | --- | --- | --- | --- |
| Anomalous data | Cook’s distance <1 | ok | ok | ok | ok | ok |
| Normality of residuals | Median ~ 0 | -.00043 | -.00037 | -.0000012 | -.0000061 | -.000006 |
|  | Histogram | ok | ok | ok | ok | ok |
|  | Shapiro-Wilk >.05 | W=.988, p=.079 | W=.993, p=.464 | W=.986, p=.031 | W=.981, p=.005 | W=.989, p=.131 |
|  | Q-Q plot | ok | ok | ok | ok | ok |
| Linearity | Plot fitted values vs residuals | ok | ok | ok | ok | ok |
|  | Plot fitted values vs residuals for each predictor | ok | ok | small dev. | deviations for stressful events | ok |
|  | Tukey test / model | -.459 p=.645 | -1.254 p=.209 | 1.286, p=.198  sig: SES (but fine on plot), Stress | -.317 p=.750  Sig: Stress | -1.111, p=.266 |
| Homogeneity of variance | Non-constant variance score >.05 | p=.051 | p=.034 | p=.137 | p=.365 | p=.853 |
| No collinearity | Variance inflation factors ~1 | 1.04-1.61 | 1.04-1.60 | 1.04-1.61 | 1.04-1.61 | 1.04-1.62 |

**Additional information for regression models**

**Frontal grey matter volume**

All assumptions for multiple regression were met. There was no anomalous data (no Cook’s distance value greater than 1). The residuals were normally distributed: the median of the residuals was -.00043 (i.e. ideally, it should be as close as possible to 0), residuals appeared normally distributed on the histogram, and the Shapiro-Wilk normality test was not significant (W=.988, p=.079). The fit line for the residuals vs fitted values plot was a relatively straight horizontal line, suggesting that the relationship is linear. There was no significant curvature for any of the predictors or the overall model (Tukey test=-.459, p=.646), and visually examining the plots suggested that the deviations from linearity are relatively small. Visual inspection of a QQ plot revealed no violations of normality assumptions. The assumption for homogeneity of variance was met (non-constant variance score p=.051). The collinearity assumption was met (all variance inflation factors were between 1.04-1.61).

**Table D. Frontal grey matter volume model A (initial model)**

| R^2^ | Adj R^2^ | F  (8,211) | p | Predictor | B | β | t | p | 95% CI |
| --- | --- | --- | --- | --- | --- | --- | --- | --- | --- |
| .42 | .40 | 19.3 | <.001 | Stress  STAI  GA  PMA  Mat age  SES  Sex(f)  Days TPN | .000018  -.000024  -.000052  .0058  -.000058  .00015  .0013  -.00010 | .067  -.016  -.007  .642  -.022  .125  .045  -.059 | 1.27  -.30  -.11  11.54  -.42  2.32  0.84  -.89 | .204  .761  .905  <.001*  .668  .020*  .401  .370 | -0.000010, 0.000046  -0.00018, 0.00013  -0.00091, 0.00080  0.0048, 0.0068  -0.00032,0.00021  0.000023,0.00027  -0.0017, 0.0044  -0.00033, 0.00012 |

We performed an exploratory analysis removing the 3 outliers from stressful life events, and the results were similar (no significant relationship with stressful life events or trait anxiety) (See RMarkdown document for details).

**Temporal grey matter volume**

For the Temporal lobe model, there was no anomalous data (no Cook’s distance value greater than 1). The residuals were normally distributed: the median of the residuals was -.00037 (i.e. ideally, it should be as close as possible to 0), residuals appeared normally distributed on the histogram, and the Shapiro-Wilk normality test was not significant (W=.993 p=.465). The fit line for the residuals vs fitted values plot was a relatively straight horizontal line, suggesting that the relationship is linear. There was no significant curvature for any of the predictors or the overall model (Tukey test=-1.254 p=.209), and visually examining the plots suggested that the deviations from linearity are relatively small. Visual inspection of a QQ plot revealed no violations of normality assumptions. The assumption for homogeneity of variance was not met (non-constant variance score p=.034). The collinearity assumption was met (all variance inflation factors were between 1.04-1.60).

As the homogeneity of variance assumption was not met, there was a chance that the standard error estimates associated with regression coefficients are no longer reliable, affecting the t and p values. Hence, we used a heteroscedasticity corrected covariance matrix when estimating standard errors. There were no substantial differences between the coefficients or their p values.

**Table E. Temporal grey matter volume model A (initial model)**

| R^2^ | Adj R^2^ | F  (8,210) | p | Predictor | B | β | t | p | 95% CI |
| --- | --- | --- | --- | --- | --- | --- | --- | --- | --- |
| .45 | .43 | 21.99 | <.001 | Stress  STAI  GA  PMA  Mat age  SES  Sex(f)  Days Vent | .0000026  .0000046  -.00026  .0025  -.000013  .000051  -.0011  -.000052 | .024  .007  -.096  .670  -.012  .104  -.094  -.072 | .469  .147  -1.514  12.40  -.237  1.98  -1.79  -1.12 | .639  .883  .131  <.001*  .813  .048*  .073  .261 | -0.0000086, 0.000014  -0.000058, 0.000067  -0.00061, 0.000080  0.0021, 0.0029  -0.00012, 0.000095  0.00000037, 0.00010  -0.0023, 0.00010  -0.00014, 0.000039 |

**Table F. Temporal grey matter volume (heteroscedasticity corrected covariance matrix)**

| Predictor | B | t | p |
| --- | --- | --- | --- |
| Stress  STAI  GA  PMA  Mat age  SES  Sex(f)  Days Vent | 0.0000026  0.0000046  -0.00026  0.0025  -0.000013  0.000051  -0.0011  -0.000052 | .49  .14  -1.26  12.39  -.24  2.09  -1.79  -.96 | .621  .888  .205  <.001*  .810  .037*  .073  .334 |

We performed an exploratory analysis removing the 3 outliers from stressful life events, and the results were similar (no significant relationship with stressful life events or trait anxiety). (See RMarkdown document for details).

**Hippocampal volume**

For the Hippocampus model, there was no anomalous data (no Cook’s distance value greater than 1). Although the Shapiro-Wilk normality test was significant (W=.981, p=.005), the median of the residuals was -.0000061 (i.e. ideally, it should be as close as possible to 0), residuals appeared relatively normally distributed on the histogram, and the QQ plot of the residuals appeared relatively linear. Given the large sample size, this analysis should be robust to violations of normality assumption. The assumption for homogeneity of variance was met (non-constant variance score p=.365). The collinearity assumption was met (all variance inflation factors were between 1.04-1.61).  Even though the Tukey test was not significant overall (Tukey test= -.318 p=.750), it suggested deviations from linearity for stressful life events (p=.028). After visual examination of plots, we removed the outliers for stressful life events and repeated the analysis. These outliers were the values 255, 264 and 270 for stressful life events. The model with outliers removed met assumptions for multiple regression (see RMarkdown document).

**Table G. Hippocampal volume models**

| R^2^ | Adj R^2^ | F | p | Predictor | B | β | t | p | 95% CI |
| --- | --- | --- | --- | --- | --- | --- | --- | --- | --- |
| MAIN MODEL | | | | | | | | | |
| .05 | .02 | 1.58 | .131 | Stress  STAI  GA  PMA  Mat age  SES  Sex(f)  Days TPN | 0.00000074  -0.0000010  -0.000012  -0.000020  -0.0000027  -0.0000040  -0.000024  0.0000012 | .11  -.03  -.08  -.09  -.04  -.14  -.03  .02 | 1.74  -.44  -.98  -1.34  -.67  -2.08  -.51  .35 | .08  .65  .32  .18  .49  .03*  .60  .72 | -0.000000098, 0.0000015  -0.0000057, 0.0000036  -0.000038, 0.000012  -0.000050, 0.0000095  -0.0000108, 0.0000053  -0.0000078, -0.00000021  -0.00011, 0.000068  -0.0000056, 0.0000081 |
| OUTLIERS REMOVED | | | | | | | | | |
| .07 | .04 | 2.17 | .030 | Stress  STAI  GA  PMA  Mat age  SES  Sex(f)  Days TPN | 0.0000012  -0.0000011  -0.000013  -0.000026  -0.0000031  -0.0000045  -0.0000081  0.0000010 | .17  -.03  -.08  -.12  -.05  -.16  -.01  .02 | 2.56  -.48  -1.01  -1.73  -.76  -2.36  -.17  .297 | .010*  .625  .313  .083  .443  .019*  .863  .766 | 0.00000028, 0.0000021  -0.0000058, 0.0000035  -0.000038, 0.000012  -0.000057, 0.0000036  -0.000011, 0.0000049  -0.0000084, -.00000075  -0.00010, 0.000085  -0.0000058, 0.0000079 |

**Amygdala volume**

For the Amygdala model, there was no anomalous data (no Cook’s distance value greater than 1). Although the Shapiro-Wilk normality test was significant (W=.986,p=.031), the median of the residuals was -.0000013 (i.e. ideally, it should be as close as possible to 0), residuals appeared relatively normally distributed on the histogram, and the QQ plot of the residuals appeared relatively linear. Given the large sample size, this analysis should be robust to violations of normality assumption. The assumption for homogeneity of variance was met (non-constant variance score p=.137). The collinearity assumption was met (all variance inflation factors were between 1.04-1.61). The fit line for the residuals vs fitted values plot was a relatively straight horizontal line, suggesting that the relationship is linear. Examining this relationship for each individual predictor revealed that the curvature was significant for stressful life events (p=.020), and socioeconomic status (p=.040), but not for the overall model (Tukey test=1.287, p=.198). Visual inspection of plots suggested that values for socioeconomic status did not contain obvious outliers. As the Tukey test suggested deviations from linearity for stressful life events, we removed the outliers and repeated the analysis. These outliers were the values 255, 264 and 270 for stressful life events. The results of the model were similar (see Table below) and met assumptions for multiple regression (see RMarkdown document for details).

**Table H. Amygdala volume regression models**

| R^2^ | Adj R^2^ | F | p | Predictor | B | β | t | p | 95% CI |
| --- | --- | --- | --- | --- | --- | --- | --- | --- | --- |
| MAIN | | | | | | | | | |
| .27 | .24 | 9.77 | <.001 | Stress  STAI  GA  PMA  Mat age  SES  Sex(f)  Days TPN | -0.000000027  -0.0000013  0.0000053  -0.000064  -0.0000024  -0.0000023  -0.000047  0.0000022 | -.0068  -.060  .054  -.467  -.064  -.129  -.109  .086 | -.114  -1.000  .735  -7.485  -1.068  -2.123  -1.799  1.159 | .909  .318  .462  <.001*  .287  .034*  .073  .247 | -0.00000050,0.00000044  -0.0000039, 0.0000012  -0.0000090, 0.000019  -0.000081, -0.000047  -0.0000070, 0.0000020  -0.0000044, 0.00000016  -0.000099, 0.0000045  -0.0000015, 0.0000061 |
| OUTLIERS REMOVED | | | | | | | | | |
| .29 | .26 | 10.72 | <.001 | Stress  STAI  GA  PMA  Mat age  SES  Sex(f)  Days TPN | 0.00000021  -0.0000013  0.0000045  -0.000068  -0.0000025  -0.0000026  -0.000038  0.0000020 | .049  -.060  .046  -.489  -.064  -.148  -.086  .078 | .083  -1.007  .629  -7.924  -1.078  -2.453  -1.435  1.052 | .404  .315  .530  <.001*  .282  .015  .153  .294 | -0.00000029,0.00000073  -0.0000039, 0.0000012  -0.0000098, 0.000019  -0.000085, -0.000051  -0.0000070, 0.0000020  -0.0000048, -0.00000052  -0.000090, 0.000014  -0.0000017, 0.0000059 |

**Thalamus volume**

All assumptions were met for the main model. For the Thalamus model, there was no anomalous data (no Cook’s distance value greater than 1). The residuals were normally distributed: the median of the residuals was -.000006 (i.e. ideally, it should be as close as possible to 0), residuals appeared normally distributed on the histogram, and the Shapiro-Wilk normality test was not significant (W=.989, p=.131). The fit line for the residuals vs fitted values plot was a relatively straight horizontal line, suggesting that the relationship is linear. There was no significant curvature for any of the predictors or the overall model (Tukey test=-1.112, p=.266), and visually examining the plots suggested that the deviations from linearity are relatively small. Visual inspection of a QQ plot revealed no violations of normality assumptions. The assumption for homogeneity of variance was met (non-constant variance score p=.853). The collinearity assumption was met (all variance inflation factors were between 1.04-1.63). Overall, all assumptions for multiple regression were met.

**Table I. Thalamus volume model**

| R^2^ | Adj R^2^ | F | p | Predictor | B | β | t | p | 95% CI |
| --- | --- | --- | --- | --- | --- | --- | --- | --- | --- |
| .08 | .04 | 2.404 | .016 | Stress  STAI  GA  PMA  Mat age  SES  Sex(f)  Days TPN | -0.00000020  -0.00000066  -0.000069  -0.0000099  -0.000015  -0.0000050  0.00050  0.000034 | -.006  -.003  -.092  -.009  -.052  -.037  .153  .172 | -.101  -.059  -1.110  -.138  -.779  -.545  2.242  2.044 | .920  .953  .268  .890  .436  .586  .026  .042 | -0.0000042, 0.0000038  -0.000023, 0.000021  -0.00019, 0.000054  -0.00015, 0.00013  -0.000054, 0.000023  -0.000023, 0.000013  0.000061, 0.00095  0.0000012, 0.000067 |

We performed an exploratory analysis removing the 3 outliers from stressful life events, and the results were similar (no significant relationship with stressful life events or trait anxiety) (See RMarkdown document for details).

We repeated all analyses subdividing the sample by sex. There were no significant relationships between maternal trait anxiety/stressful events and infant volume in frontal lobe, temporal lobe, amygdala, thalamus. (See RMarkdown code for details).

**Additional information on anxiety and stressful life events**

For the trait anxiety measure, a cut-off of 40 indicates that a woman may have an anxiety disorder [1]. In our sample n=88 women scored 40 or over (n=60 scored 40-49, n=23 scored 50-59, and n=5 scored 60-69) and n=133 scored under 40 (n=85 scored 30-39 and n=48 scored 20-29). The median of the Trait anxiety subscale of the STAI (median =36) was below clinical cut-offs [2, 3], and previous studies reporting associations between maternal anxiety and brain development included participants with higher levels of anxiety (e.g. threshold of 43 in [4]). It is important to acknowledge the possibility that focusing on trait anxiety rather than state anxiety could have influenced our findings. While some previous studies in infants have reported differences in brain development related to maternal state anxiety [5] or a combined score of state and trait anxiety [4], these studies analysed white matter microstructure using diffusion MRI. To our knowledge, the only other study which has used the STAI to assess differences in brain volume in infants [6], only included trait anxiety measures. Their results were in line with our current findings, as they reported that maternal trait anxiety was not associated with any differences in hippocampal volume at birth.

Our decision to study trait anxiety instead of state anxiety is motivated by the fact that trait anxiety is considered to be a relatively stable personality trait, while state anxiety reflects a transient anxious state. State anxiety scores were not included in the analysis, as this measure was designed to be sensitive to the conditions under which the questionnaire is administered [7]. It is likely that state anxiety scores would have been influenced by the stress-inducing situations of having a preterm baby, being in a hospital environment, and attending an appointment for an infant MRI scan, and thus would not be representative of prenatal anxiety levels.

In contrast, trait anxiety scores imply a generalized and enduring predisposition to respond to situations in an anxious manner, are less likely to be influenced by situational variables [7] and are thus more likely to be reflective of the mother’s prenatal anxiety levels. In the perinatal period, studies have reported that self-reported trait anxiety is not a transient state, but is relatively stable between pregnancy and 7 months postpartum [3].

With regards to the reliability of recall for life events, studies report that false positive reports of adverse life events are rare [8], and that for pregnancy-related events, there is high reliability between data collected during pregnancy, and self-report measures collected up to 30 years after birth [9, 10].

**Additional information regarding the stressful life events measure**

A detailed description of the stressful life events measure can be read in [11]. The list of events and the frequency of responses per each item can be seen in Table S10. In our sample, the number of stressful life events experienced by the participants ranged from 0-7 (with a mean of 1.8, and a standard deviation of 1.34). The final score per participant was created by summing the severity scores for each item experienced (median=53, range=0-270). The final stressful life events scores for this sample were similar to those in [11].

**Table J. Frequency of responses and severity scores for Stressful Life Events.**

| Stressful life event item | N= | Severity score |
| --- | --- | --- |
| You were separated or divorced from your partner (item 6) | 12 | 69 |
| You had a serious illness of injury (item 1) | 15 | 53 |
| You were physically assaulted (item 18) | 2 | 53 |
| You had a miscarriage (item 19) | 34 | 53 |
| A family member or close friend died (item 2) | 41 | 50 |
| You lost your job (item 10) | 7 | 47 |
| A family member or close friend was seriously ill (item 3) | 32 | 44 |
| You had treatment for infertility (item 20) | 41 | 44 |
| Your house was burgled (item 17) | 3 | 40 |
| You had a major financial problem (item 15) | 16 | 38 |
| You were in trouble with the law (item 4) | 0 | 37 |
| Your partner lost his/her job (item 11) | 17 | 37 |
| Arguments with your partner increased (item 12) | 34 | 35 |
| You changed jobs (item 9) | 19 | 33 |
| You had a serious argument with family or friends (item 13) | 14 | 29 |
| Your partner was in trouble with the law (item 5) | 2 | 27 |
| You moved to a new house/new place to live (item 14) | 65 | 25 |
| You had a personal problem at work (item 7) | 17 | 23 |
| You took an examination (item 16) | 10 | 19 |
| Your partner had problems at work (item 8) | 13 | 13 |

**Additional information regarding the structures being investigated**

Overall, the literature has suggested that maternal prenatal stress affects brain development primarily in the frontal lobe, temporal lobe, and the limbic system. These are areas that are also affected in a range of psychiatric and neurodevelopmental disorders, and altered development of these areas may play a role in the adverse outcomes associated to maternal prenatal stress. However, these changes are not reported consistently between studies, and there are several inconsistencies that need addressing. It is as of yet unclear whether brain alterations related to prenatal maternal depression and/or anxiety are restricted to one or several brain regions, or whether changes are non-specific and differ between infants based on a combination of genetic and early developmental factors. Here, we summarise some of the existing literature related to the regions that were selected for our main analysis. As we acknowledge that the effects of maternal stress are often heterogeneous, we supplemented our region of interest analysis with a whole-brain analysis using Jacobian determinants. See below for a short summary of literature related to the chosen regions of interest.

**The frontal lobe**

The frontal lobes are functionally heterogeneous, with involvement in a wide range of aspects, such as motor skills, emotional regulation, impulse control, attention, reasoning, and problem solving [12].

Differences in the frontal lobe are the most consistently reported structural imaging findings in human studies of maternal stress. However, it is unclear whether certain sub-areas of the frontal lobe are particularly vulnerable to stress. Prenatal stress studies have reported volume differences in the prefrontal cortex [13], specifically the right superior, medial orbital and frontal pole regions [14], pars opercularis [15, 16], pars triangularis and precentral and rostral middle frontal regions [16]. Other regions of the frontal lobe reported in the prenatal stress literature include the premotor cortex [13], paracentral cortex [15], postcentral gyrus [13], left caudal middle frontal area [17], superior frontal lobes [15, 17], and anterior cingulate cortex [18].

Volume abnormalities in various regions of the frontal lobe have also been widely reported in a range of psychiatric disorders, such as depression and anxiety [19-21], as well as in those exposed to childhood maltreatment or adversity [22].

**Temporal lobe**

The function of the temporal lobe is often discussed in the context of memory [23], and attention [24], but volume abnormalities in regions of the temporal lobe have been reported in mood disorders [20,21], children exposed to maltreatment [22], and adults who experienced parental verbal abuse during childhood [25]. In the context of maternal prenatal stress, studies have also reported differences in temporal lobe volume, including the medial [13, 16, 26] and lateral [13] regions. A study in a fetal sample reported increased gyrification in frontal and temporal lobes [27].

**The amygdala**

The function of the amygdala is primarily discussed in the context of evaluating the emotional significance of stimuli, and is believed to be involved in the perception of threat and fear, as well as the bias towards a negative emotional valence in depression [28].

Differences in amygdala volumes are commonly reported in a range of psychiatric disorders and exposure to early environmental stressors. For example, differences in amygdala volume have been reported in depression [29, 30], early deprivation or maltreatment [22, 25, 31, 32] and trauma and anxiety [33, 34]. In one study, children who were exposed to maternal depression since birth had larger bilateral amygdala volumes compared to those who were not exposed [35].

The direction of effect is often unclear and inconsistent. Amygdala volumes have been found to be normal, enlarged, and decreased in chronically depressed adults [36] and those exposed to maltreatment [25]. It has been suggested that early life stress causes an initial increase in amygdala volume, but also causes the amygdala to be more sensitive to subsequent stressors, leading to a graded reduction in volume [37, 38].

In the context of maternal prenatal stress, one of the most consistently reported findings from structural MRI studies is represented by volume differences in the amygdala [26, 39-42].

**Hippocampus**

The function of the hippocampus is believed to be largely related to long-term memory [43], but differences in hippocampal volume are often reported in studies of depression and anxiety [19, 30, 44, 21] and early adversity and maltreatment [22, 25]. Smaller hippocampal volumes are reported in children of mothers with a history of depression and those identified as being at high risk for depression [45]. Studies have suggested that hippocampal volume changes are evident in those exposed to adversity, even prior to, or in the absence of psychopathology, and have suggested that hippocampal volume changes arise as a combination of adversity and genetic vulnerability [25, 43]. In the context of maternal prenatal stress, a few studies have reported differences in hippocampal volume [46, 27].

**Thalamus**

The thalamus is considered an essential hub for information transfer and integration [47], and its function is often discussed in the context of emotion, memory, and arousal, with volume differences reported in those with mood and anxiety disorders [19], and those exposed to early adversity [48]. In one study of infants born very preterm, the volume of the thalamus was related to neonatal pain-related stress [49].

**Prematurity and brain development**

In a healthy intrauterine environment, the later half of the second trimester and the first half of the third trimester are characterised by a wide range of complex and interrelated events, such as neuronal migration, glial cell proliferation, and synapse formation [50]. In terms of macrostructural development, the gyri and sulci begin to form around 8 weeks of gestation, the central sulcus is defined by 27 weeks, and the majority of gyri and sulci are developed by 37 weeks. For the frontal and temporal lobe, there is minimal folding around 31 weeks, with gyral and sulcal development being evident around 34 weeks [50].

Brain development is disrupted by premature birth, often in a regionally specific manner. For example, volume reductions in cortical grey matter [51-53] and deep grey matter [54] are commonly reported in infants born prematurely. Therefore, it is important to understand whether maternal prenatal stress affects brain development above and beyond any impact of prematurity.

**Sensitivity analysis : Days on ventilation**

“Days on total parenteral nutrition (TPN)” was included in the model over “days on ventilation” based on the distribution of scores (Days TPN: median=6, range 0-59; Days Ventilation: median=0, range 0-33), as well as previous literature [11].

We repeated our main analysis including days on ventilation in the model, instead of days on TPN, to assess whether this change would affect the results. The pattern of results remained the same. More specifically, for stressful life events, there was no association with frontal lobe volume (B=.000017, t=1.19, p=.234), temporal lobe volume (B=.0000017, t=.297, p=.766), thalamus volume (B=-.00000030, t=-.14, p=.884), amygdala volume (B=-.000000028, t=-.11, p=.906), or hippocampal volume (B=.00000068, t=1.59, p=.112). For trait anxiety, there was no association with frontal lobe volume (B=-.000029, t=-.36, p=.713), temporal lobe volume (B=.0000037, t=0.11, p=.905), thalamus volume (B=.0000026, t=0.23, p=.817), amygdala volume (B=-.0000011, t=-.85, p=.395), or hippocampal volume (B=-.00000070, t=-.29, p=.765).

**CONTRIBUTOR ROLES TAXONOMY**

The Contributor Roles Taxonomy (CRediT) is a consensus-based classification system that distinguishes different contributor roles. Authors have been listed next to each role they contributed towards.

Initials are as follows: Alexandra Lautarescu (AL), Laila Hadaya (LH), Michael C. Craig (MCC), Antonis Makropoulos (AM), Dafnis Batalle (DB), Chiara Nosarti (CN), A. David Edwards (ADE), Serena J. Counsell (SJC), Suresh Victor (SV).

Table K. Contributor role taxonomy.

| **Role** | **Definition** | **Authors** |
| --- | --- | --- |
| Conceptualization | Ideas; formulation or evolution of overarching research goals and aims | AL, MCC, ADE, SJC, SV, CN |
| Data curation | Management activities to annotate (produce metadata), scrub data and maintain research data (including software code, where it is necessary for interpreting the data itself) for initial use and later re-use | AL, LH |
| Formal analysis | Application of statistical, mathematical, computational, or other formal techniques to analyse or synthesize study data. | AL, LH, AM,DB |
| Funding acquisition | Acquisition of the financial support for the project leading to this publication. | ADE, SJC |
| Methodology | Development or design of methodology; creation of models. | AM |
| Project administration | Management and coordination responsibility for the research activity planning and execution. | ADE, SJC |
| Resources | Provision of study materials, reagents, materials, patients, laboratory samples, animals, instrumentation, computing resources, or other analysis tools. | ADE, SJC |
| Software | Programming, software development; designing computer programs; implementation of the computer code and supporting algorithms; testing of existing code components. | AM, DB |
| Supervision | Oversight and leadership responsibility for the research activity planning and execution, including mentorship external to the core team. | MCC, SJC, SV, ADE, CN, DB |
| Validation | Verification, whether as a part of the activity or separate, of the overall replication/reproducibility of results/experiments and other research outputs. | AL |
| Visualization | Preparation, creation and/or presentation of the published work, specifically visualization/data presentation | AL, LH |
| Writing -original draft | Preparation, creation and/or presentation of the published work, specifically writing the initial draft (including substantive translation) | AL, LH |
| Writing – review & editing | Preparation, creation and/or presentation of the published work by those from the original research group, specifically critical review, commentary or revision – including pre- or post-publication stages. | AL, LH, MCC, AM, DB, CN, ADE, SJC, SV |

**References**

1. Sinesi A, Maxwell M, O'Carroll R, Cheyne H. Anxiety scales used in pregnancy: systematic review. BJPsych open. 2019 Jan;5(1).

2. Dennis CL, Coghlan M, Vigod S. Can we identify mothers at-risk for postpartum anxiety in the immediate postpartum period using the State-Trait Anxiety Inventory?. Journal of Affective Disorders. 2013 Sep 25;150(3):1217-20.

3. Grant KA, McMahon C, Austin MP. Maternal anxiety during the transition to parenthood: a prospective study. Journal of affective disorders. 2008 May 1;108(1-2):101-11.

4. Rifkin-Graboi A, Meaney MJ, Chen H, Bai J, Hameed WB, Tint MT et al. Antenatal maternal anxiety predicts variations in neural structures implicated in anxiety disorders in newborns. Journal of the American Academy of Child & Adolescent Psychiatry. 2015 Apr 1;54(4):313-21.

5. Dean DC, Planalp EM, Wooten W, Kecskemeti SR, Adluru N, Schmidt CK et al. Association of prenatal maternal depression and anxiety symptoms with infant white matter microstructure. JAMA pediatrics, 2018. 172(10), 973-981.

6. Qiu A, Rifkin-Graboi A, Chen H, Chong YS, Kwek K, Gluckman PD et al. Maternal anxiety and infants' hippocampal development: timing matters. Translational psychiatry. 2013 Sep;3(9):e306-.

7. Spielberger CD. Anxiety as an emotional state. Anxiety-Current trends and theory. 1972:3-20.

8. Hardt J, Rutter M. Validity of adult retrospective reports of adverse childhood experiences: review of the evidence. Journal of child psychology and psychiatry. 2004 Feb;45(2):260-73.

9. Tomeo CA, Rich-Edwards JW, Michels KB, Berkey CS, Hunter DJ, Frazier AL et al. Reproducibility and validity of maternal recall of pregnancy-related events. Epidemiology. 1999 Nov 1:774-7.

10. Quigley MA, Hockley C, Davidson LL. Agreement between hospital records and maternal recall of mode of delivery: evidence from 12 391 deliveries in the UK Millennium Cohort Study. BJOG: An International Journal of Obstetrics & Gynaecology. 2007 Feb;114(2):195-200.

11. Lautarescu A, Pecheva D, Nosarti C, Nihouarn J, Zhang H, Victor S et al. Maternal prenatal stress is associated with altered uncinate fasciculus microstructure in premature neonates. Biological psychiatry. 2020., 87(6), 559-569

12. Scott JG, Schoenberg MR. Frontal lobe/executive functioning. InThe little black book of neuropsychology 2011 (pp. 219-248). Springer, Boston, MA.

13. Buss C, Davis EP, Muftuler LT, Head K, Sandman CA. High pregnancy anxiety during mid-gestation is associated with decreased gray matter density in 6–9-year-old children. Psychoneuroendocrinology. 2010 Jan 1;35(1):141-53.

14. Sandman CA, Buss C, Head K, Davis EP. Fetal exposure to maternal depressive symptoms is associated with cortical thickness in late childhood. Biological psychiatry. 2015 Feb 15;77(4):324-34.

15. Davis EP, Hankin BL, Glynn LM, Head K, Kim DJ, Sandman CA. Prenatal maternal stress, child cortical thickness, and adolescent depressive symptoms. Child development. 2020 Mar;91(2):e432-50.

16. Lebel C, Walton M, Letourneau N, Giesbrecht GF, Kaplan BJ, Dewey D. Prepartum and postpartum maternal depressive symptoms are related to children’s brain structure in preschool. Biological psychiatry. 2016 Dec 1;80(11):859-68.

17. El Marroun H, Tiemeier H, Muetzel RL, Thijssen S, van der Knaap NJ, Jaddoe VW et al. Prenatal exposure to maternal and paternal depressive symptoms and brain morphology: A population‐based prospective neuroimaging study in young children. Depression and anxiety. 2016 Jul;33(7):658-66.

18. Marečková K, Klasnja A, Bencurova P, Andrýsková L, Brázdil M, Paus T. Prenatal stress, mood, and gray matter volume in young adulthood. Cerebral Cortex. 2019 Mar 1;29(3):1244-50.

19. Zhang FF, Peng W, Sweeney JA, Jia ZY, Gong QY. Brain structure alterations in depression: psychoradiological evidence. CNS neuroscience & therapeutics. 2018 Nov;24(11):994-1003.

20. Price JL, Drevets WC. Neurocircuitry of mood disorders. Neuropsychopharmacology. 2010 Jan;35(1):192-216.

21. Beyer JL, Krishnan KR. Volumetric brain imaging findings in mood disorders. Bipolar Disorders. 2002 Apr;4(2):89-104.

22. Bick J, Nelson CA. Early adverse experiences and the developing brain. Neuropsychopharmacology. 2016 Jan;41(1):177-96.

23. Shimotake A, Matsumoto R, Ueno T, Kunieda T, Saito S, Hoffman P et al. Direct exploration of the role of the ventral anterior temporal lobe in semantic memory: cortical stimulation and local field potential evidence from subdural grid electrodes. Cerebral Cortex. 2015 Oct 1;25(10):3802-17.

24. Rubia K, Smith AB, Brammer MJ, Taylor E. Temporal lobe dysfunction in medication-naive boys with attention-deficit/hyperactivity disorder during attention allocation and its relation to response variability. Biological psychiatry. 2007 Nov 1;62(9):999-1006.

25. Teicher MH, Samson JA, Anderson CM, Ohashi K. The effects of childhood maltreatment on brain structure, function and connectivity. Nature Reviews Neuroscience. 2016 Oct;17(10):652.

26. Favaro A, Tenconi E, Degortes D, Manara R, Santonastaso P. Neural correlates of prenatal stress in young women. Psychological medicine. 2015 Sep 1;45(12):2533.

27. Wu Y, Lu YC, Jacobs M, Pradhan S, Kapse K, Zhao L et al. Association of Prenatal Maternal Psychological Distress With Fetal Brain Growth, Metabolism, and Cortical Maturation. JAMA network open. 2020 Jan 3;3(1):e1919940-.

28. Bellani M, Baiano M, Brambilla P. Brain anatomy of major depression II. Focus on amygdala. Epidemiology and psychiatric sciences. 2011 Mar;20(1):33-6.

29. Bora E, Fornito A, Pantelis C, Yücel M. Gray matter abnormalities in major depressive disorder: a meta-analysis of voxel based morphometry studies. Journal of affective disorders. 2012 Apr 1;138(1-2):9-18.

30. Schmaal L, Veltman DJ, van Erp TG, Sämann PG, Frodl T, Jahanshad N et al. Subcortical brain alterations in major depressive disorder: findings from the ENIGMA Major Depressive Disorder working group. Molecular psychiatry. 2016 Jun;21(6):806-12.

31. Mehta MA, Golembo NI, Nosarti C, Colvert E, Mota A, Williams SC et al. Amygdala, hippocampal and corpus callosum size following severe early institutional deprivation: the English and Romanian Adoptees study pilot. Journal of Child Psychology and Psychiatry. 2009 Aug;50(8):943-51.

32. Tottenham N, Hare TA, Quinn BT, McCarry TW, Nurse M, Gilhooly T et al. Prolonged institutional rearing is associated with atypically large amygdala volume and difficulties in emotion regulation. Developmental science. 2010 Jan;13(1):46-61.

33. Weems CF, Russell JD, Neill EL, McCurdy BH. Annual research review: Pediatric posttraumatic stress disorder from a neurodevelopmental network perspective. Journal of Child Psychology and Psychiatry. 2019 Apr;60(4):395-408.

34. De Bellis MD, Casey BJ, Dahl RE, Birmaher B, Williamson DE, Thomas KM et al. A pilot study of amygdala volumes in pediatric generalized anxiety disorder. Biological psychiatry. 2000 Jul 1;48(1):51-7.

35. Lupien SJ, Parent S, Evans AC, Tremblay RE, Zelazo PD, Corbo V et al. Larger amygdala but no change in hippocampal volume in 10-year-old children exposed to maternal depressive symptomatology since birth. Proceedings of the National Academy of Sciences. 2011 Aug 23;108(34):14324-9.

36. Hulvershorn LA, Cullen K, Anand A. Toward dysfunctional connectivity: a review of neuroimaging findings in pediatric major depressive disorder. Brain imaging and behavior. 2011 Dec 1;5(4):307-28.

37. Kuo JR, Kaloupek DG, Woodward SH. Amygdala volume in combat-exposed veterans with and without posttraumatic stress disorder: a cross-sectional study. Archives of general psychiatry. 2012 Oct 1;69(10):1080-6.

38. Hanson JL, Nacewicz BM, Sutterer MJ, Cayo AA, Schaefer SM, Rudolph KD et al. Behavioral problems after early life stress: contributions of the hippocampus and amygdala. Biological psychiatry. 2015 Feb 15;77(4):314-23.

39. Lugo-Candelas C, Cha J, Hong S, Bastidas V, Weissman M, Fifer WP et al. Associations between brain structure and connectivity in infants and exposure to selective serotonin reuptake inhibitors during pregnancy. JAMA pediatrics. 2018 Jun 1;172(6):525-33.

40. Wen DJ, Poh JS, Ni SN, Chong YS, Chen H, Kwek K et al. Influences of prenatal and postnatal maternal depression on amygdala volume and microstructure in young children. Translational psychiatry. 2017 Apr;7(4):e1103-.

41. Acosta H, Tuulari JJ, Scheinin NM, Hashempour N, Rajasilta O, Lavonius TI et al. Maternal pregnancy-related anxiety is associated with sexually dimorphic alterations in amygdala volume in four-year-old children. Frontiers in behavioral neuroscience. 2019;13:175.

42. Jones SL, Dufoix R, Laplante DP, Elgbeili G, Patel R, Chakravarty MM et al. Larger amygdala volume mediates the association between prenatal maternal stress and higher levels of externalizing behaviors: sex specific effects in project ice storm. Frontiers in human neuroscience. 2019 May 14;13:144.

43. Rajmohan V, Mohandas E. The limbic system. Indian journal of psychiatry. 2007 Apr;49(2):132.

44. Kempton MJ, Salvador Z, Munafo MR, Geddes JR, Simmons A, Frangou S et al. Structural neuroimaging studies in major depressive disorder: meta-analysis and comparison with bipolar disorder. Archives of general psychiatry. 2011 Jul 4;68(7):675-90.

45. Chen MC, Hamilton JP, Gotlib IH. Decreased hippocampal volume in healthy girls at risk of depression. Archives of general psychiatry. 2010 Mar 1;67(3):270-6.

46. Rao U, Chen LA, Bidesi AS, Shad MU, Thomas MA, Hammen CL. Hippocampal changes associated with early-life adversity and vulnerability to depression. Biological psychiatry. 2010 Feb 15;67(4):357-64.

*47*. Hwang K, Bertolero MA, Liu WB, D'esposito M. The human thalamus is an integrative hub for functional brain networks. Journal of Neuroscience. 2017 Jun 7;37(23):5594-607.

48. Liao M, Yang F, Zhang Y, He Z, Song M, Jiang T et al. Childhood maltreatment is associated with larger left thalamic gray matter volume in adolescents with generalized anxiety disorder. PLoS One. 2013 Aug 12;8(8):e71898.

49. Chau CM, Ranger M, Bichin M, Park MT, Amaral RS, Chakravarty M et al. Hippocampus, amygdala, and thalamus volumes in very S children at 8 years: neonatal pain and genetic variation. Frontiers in behavioral neuroscience. 2019 Mar 19;13:51

50. Peterson BS. Brain imaging studies of the anatomical and functional consequences of preterm birth for human brain development. Annals of the New York Academy of Sciences. 2003 Dec;1008(1):219-37.

51. Nosarti C, Al‐Asady MH, Frangou S, Stewart AL, Rifkin L, Murray RM. Adolescents who were born very preterm have decreased brain volumes. Brain. 2002 Jul 1;125(7):1616-23.

52. Vinall J, Grunau RE, Brant R, Chau V, Poskitt KJ, Synnes AR et al. Slower postnatal growth is associated with delayed cerebral cortical maturation in preterm newborns. Science translational medicine. 2013 Jan 16;5(168):168ra8-.

53. Inder TE, Huppi PS, Warfield S, Kikinis R, Zientara GP, Barnes PD et al. Periventricular white matter injury in the premature infant is followed by reduced cerebral cortical gray matter volume at term. Annals of Neurology: Official Journal of the American Neurological Association and the Child Neurology Society. 1999 Nov;46(5):755-60.

54. Boardman JP, Counsell SJ, Rueckert D, Kapellou O, Bhatia KK, Aljabar P et al. Abnormal deep grey matter development following preterm birth detected using deformation-based morphometry. Neuroimage. 2006 Aug 1;32(1):70-8.
