# Supplementary material for: Exploring the relationship between maternal prenatal stress and brain structure in premature neonates
Source: PLoS One. 2021 Apr 21;16(4):e0250413. doi: 10.1371/journal.pone.0250413 (PMC8059832; doi:10.1371/journal.pone.0250413)
Supplement: Attachment — Submitted filename: Response to Reviewers.docx [file pone.0250413.s004.docx]

**Response to Reviewers**

Journal requirements:

When submitting your revision, we need you to address these additional requirements.

1. Please ensure that your manuscript meets PLOS ONE's style requirements, including those for file naming. The PLOS ONE style templates can be found at

https://journals.plos.org/plosone/s/file?id=wjVg/PLOSOne_formatting_sample_main_body.pdf and

https://journals.plos.org/plosone/s/file?id=ba62/PLOSOne_formatting_sample_title_authors_affiliations.pdf

 Author’s response: Journal requirements #1

We have now updated our manuscript to ensure it meets PLOS ONE’s style requirements.

2. In your Methods section, please provide additional information about the participant recruitment method and the demographic details of your participants. Please ensure you have provided sufficient details to replicate the analyses such as:
a) the recruitment date range (month and year),
b) a statement as to whether your sample can be considered representative of a larger population, and
c) a description of how participants were recruited.

Author’s response: Journal requirements #2

In response to this comment, we have added the following information to the Methods section:

“Participants were recruited between April 2010 and July 2013 by screening 3619 admissions to level 1,2 and 3 neonatal units at 14 London Hospitals. Eligibility criteria for the main study included: infant born before 33 weeks gestational age, mother aged over 16 years, not a hospital inpatient, no major congenital malformation, no prior MRI, no care in a centre where preterm MRI was routine, no metallic implants, parents able to speak English, parents not subject to child protection proceedings. The ePrime cohort is representative of the UK NICU population in terms of birth weight, ethnicity, and prevalence of cerebral palsy (6%). Additional information is available in Edwards et al. (2009)”

3. We note that you have indicated that data from this study are available upon request. PLOS only allows data to be available upon request if there are legal or ethical restrictions on sharing data publicly. For information on unacceptable data access restrictions, please see http://journals.plos.org/plosone/s/data-availability#loc-unacceptable-data-access-restrictions.

In your revised cover letter, please address the following prompts:

a) If there are ethical or legal restrictions on sharing a de-identified data set, please explain them in detail (e.g., data contain potentially identifying or sensitive patient information) and who has imposed them (e.g., an ethics committee). Please also provide contact information for a data access committee, ethics committee, or other institutional body to which data requests may be sent.

b) If there are no restrictions, please upload the minimal anonymized data set necessary to replicate your study findings as either Supporting Information files or to a stable, public repository and provide us with the relevant URLs, DOIs, or accession numbers. Please see http://www.bmj.com/content/340/bmj.c181.long for guidelines on how to de-identify and prepare clinical data for publication. For a list of acceptable repositories, please see http://journals.plos.org/plosone/s/data-availability#loc-recommended-repositories.

We will update your Data Availability statement on your behalf to reflect the information you provide.

Author’s response: Journal requirements #3

As our data contained sensitive and potentially identifiable information, we were unable to share the full anonymised dataset necessary to replicate the study findings. However, we have now shared a dataset (S3 Dataset) containing the following variables: stressful life events score, trait anxiety score, gestational age at birth, postmenstrual age at scan, socioeconomic status, sex, and brain volumes for the regions of interest.

The variables above represent the vast majority of variables included in the regression models reported in this manuscript. We were unable to share the variable “maternal age”, as this combination of variables would increase the risk of the data being identifiable. We have conducted a sensitivity analysis removing “maternal age” from the regression models, with no major differences in the results (i.e. there was still no significant relationship between maternal stress or anxiety and any of the volumes of interest).

We have now updated our data availability statement as follows:

“Relevant data have been shared within the Supporting Information files. This data includes the majority of the variables included in the regression models reported in the manuscript. As this data contained sensitive and potentially identifiable information, we were unable to share the full anonymised dataset necessary to replicate the study findings. Data for the variable "maternal age" could not be shared, as this combination of variables would increase the risk of the data being identifiable. Requests related to data access can be directed to the Hammersmith and Queen Charlotte’s Research Ethics Committee ([westlondon.rec@hra.nhs.uk)](mailto:westlondon.rec@hra.nhs.uk)).”

4. Please include captions for your Supporting Information files at the end of your manuscript, and update any in-text citations to match accordingly. Please see our Supporting Information guidelines for more information: [http://journals.plos.org/plosone/s/supporting-information](about:blank).

 Author’s response: Journal requirements #4

We thank the editor for this comment. We have now updated the captions for our supporting information files at the end of the manuscript, and have updated all in-text citations to match accordingly.

Reviewers' comments:

Reviewer's Responses to Questions

**Comments to the Author**

1. Is the manuscript technically sound, and do the data support the conclusions?

The manuscript must describe a technically sound piece of scientific research with data that supports the conclusions. Experiments must have been conducted rigorously, with appropriate controls, replication, and sample sizes. The conclusions must be drawn appropriately based on the data presented.

Reviewer #1: Yes

Reviewer #2: Yes

Author’s response #1

We thank the reviewers for their comments.

2. Has the statistical analysis been performed appropriately and rigorously?

Reviewer #1: Yes

Reviewer #2: Yes

Author’s response #2

We thank the reviewers for their comments.

3. Have the authors made all data underlying the findings in their manuscript fully available?

The [PLOS Data policy](https://eur03.safelinks.protection.outlook.com/?url=http%3A%2F%2Fwww.plosone.org%2Fstatic%2Fpolicies.action%23sharing&data=04%7C01%7Calexandra.lautarescu%40kcl.ac.uk%7C5a1f561de88944cf265808d8c24b5483%7C8370cf1416f34c16b83c724071654356%7C0%7C0%7C637472976113192814%7CUnknown%7CTWFpbGZsb3d8eyJWIjoiMC4wLjAwMDAiLCJQIjoiV2luMzIiLCJBTiI6Ik1haWwiLCJXVCI6Mn0%3D%7C1000&sdata=a0uNR4tUZu7y%2B2%2BE5kcjro4XR%2Bx%2BPVjkmPTjjQ%2FVf6g%3D&reserved=0) requires authors to make all data underlying the findings described in their manuscript fully available without restriction, with rare exception (please refer to the Data Availability Statement in the manuscript PDF file). The data should be provided as part of the manuscript or its supporting information, or deposited to a public repository. For example, in addition to summary statistics, the data points behind means, medians and variance measures should be available. If there are restrictions on publicly sharing data—e.g. participant privacy or use of data from a third party—those must be specified.

Reviewer #1: Yes

Reviewer #2: No

Author’s response #3

We thank the reviewers for their comments

We have now shared a minimal de-identified data set (S3. Dataset). Please see the section “Additional information regarding data and code” in “S1 Supplement” for details regarding this dataset.

4. Is the manuscript presented in an intelligible fashion and written in standard English?

PLOS ONE does not copyedit accepted manuscripts, so the language in submitted articles must be clear, correct, and unambiguous. Any typographical or grammatical errors should be corrected at revision, so please note any specific errors here.

Reviewer #1: Yes

Reviewer #2: Yes

Author’s response #3

We thank the reviewers for their comments.

5. Review Comments to the Author

Please use the space provided to explain your answers to the questions above. You may also include additional comments for the author, including concerns about dual publication, research ethics, or publication ethics. (Please upload your review as an attachment if it exceeds 20,000 characters)

**Reviewer #1:** In this study the authors aim to find an association between maternal stress and brain volumes in areas adjacent to the uncinate fasciculus tract. They found no evidence that maternal prenatal stressful life events or trait anxiety influence volumes in the hippocampus, amygdala, thalamus, frontal grey matter or temporal grey matter volume.
The study is well designed and even if complex to understand it is well written and it is interesting to the field.
This reviewer would want to raise some concerns:
The authors would maybe want to discuss a bit further how studying trait anxiety instead of state anxiety could have driven the findings of non-association. While the authors acknowledge this in the discussion (page 17, lines 424) the first line of the introduction talks about poor maternal health during pregnancy and could prone the reader, non-expert in STAI, to confusion.

Author’s response: Reviewer 1 Comment 1

We thank the reviewer for this insightful comment.

Firstly, we would like to draw the reviewer’s attention to S1 Supplement – Supporting Information, where we have included a section on “Additional information on anxiety and stressful life events”, which we hope motivates our decision to study trait anxiety instead of state anxiety.

To further clarify, we have added the following paragraph to S1 Supplement:

“It is important to acknowledge the possibility that focusing on trait anxiety rather than state anxiety could have influenced our findings. While some previous studies in infants have reported differences in brain development related to maternal state anxiety (Dean et al., 2018) or a combined score of state and trait anxiety (Rifkin-Graboi et al., 2015), these studies analysed white matter microstructure using diffusion MRI. To our knowledge, the only other study which has used the STAI to assess differences in brain volume in infants (Qiu et al., 2013), only included trait anxiety measures. Their results were in line with our current findings, as they reported that maternal trait anxiety was not associated with any differences in hippocampal volume at birth.

Our decision to study trait anxiety instead of state anxiety is motivated by the fact that trait anxiety is considered to be a relatively stable personality trait, while state anxiety reflects a transient anxious state. State anxiety scores were not included in the analysis, as this measure was designed to be sensitive to the conditions under which the questionnaire is administered (Spielberger, 1972). It is likely that state anxiety scores would have been influenced by the stress-inducing situations of having a preterm baby, being in a hospital environment, and attending an appointment for an infant MRI scan, and thus would not be representative of prenatal anxiety levels.

In contrast, trait anxiety scores imply a generalized and enduring predisposition to respond to situations in an anxious manner, are less likely to be influenced by situational variables (Spielberger et al., 1972) and are thus more likely to be reflective of the mother’s prenatal anxiety levels. In the perinatal period, studies have reported that self-reported trait anxiety is not a transient state, but is relatively stable between pregnancy and 7 months postpartum (Grant et al., 2008). “

We would also like to draw the reviewer’s attention to our Methods section, where we have included the sentence “The analysis was restricted to trait anxiety, as it measures a relatively stable tendency to be prone to experiencing anxiety and thus extends to the period before birth “. To further emphasize this point, we have now also added the following to the Discussion section of the manuscript: “self-reported trait anxiety scores are relatively stable in the perinatal period (Grant et al., 2008) (See S1 Supplement for further discussion).”

Lastly, to avoid any confusion for the reader, we have added further clarifications in the manuscript and replaced multiple instances of “anxiety” with “trait anxiety”.

Based on the number of days on parenteral nutrition being correlated with the number of days on mechanical ventilation the authors exclude the latter which is surprising for this reviewer , as based on the theoretical background and the literature on the impact of duration of mechanical ventilation and bronchopulmonary dysplasia on cognitive outcomes one might speculate that duration on mechanical ventilation would have a bigger effect size on brain volumes than that of duration of parenteral nutrition. The argument the authors describe as to exclude this “both provide information on the health status of the infant” seems to reflect an arbitrary decision instead of a decision based on some preliminary analysis of the effect of both variables.

Author’s response: Reviewer 1 Comment 2

We thank the reviewer for the opportunity to further clarify our analytical decision.

We would like to reassure the reviewer that our decision to include “days on parenteral nutrition” in the model instead of “days on mechanical ventilation” was not arbitrary, but was based on several reasons which we outline below.

First of all, “days on total parenteral nutrition” was included in the model over “days on ventilation” based on the distribution of scores (Days TPN: median=6, range 0-59, Days Ventilation: median=0, range 0-33), as it was less skewed. To further clarify this, we have now also added additional information to our Methods section (Statistical analysis: Main analysis) where we have followed “both measures provide information on the health status of the infant” with “and the distribution of days on total parenteral nutrition was less skewed”.

Secondly, in our previous paper using diffusion tensor imaging data on an overlapping sample (Lautarescu et al., 2020), we have used days on total parenteral nutrition in the model, based on its association with uncinate fasciculus microstructure. The regression models were the same for the two papers, and our belief is that the consistency in methodology helps strengthen our argument. This is highlighted in our manuscript (“The regression models used were the same as those used in Lautarescu et al., 2020”).

In Lautarescu et al., 2020, we have performed numerous sensitivity analyses to check the robustness of the results, and reported that the pattern of results remained the same when including days of ventilation in the model instead of days on total parenteral nutrition. Because of this, we felt confident in conducting the analysis on brain volumes using the same statistical models (i.e. including total parenteral nutrition in the model).

Following the reviewer’s comment, we have repeated our main analysis using days on ventilation in the model, instead of days on parenteral nutrition. The pattern of results remained the same, with no significant association between maternal stressful life events or maternal trait anxiety, and any of the brain volumes of interest. We report a summary of the results of this sensitivity analysis in S1 Supplement (Sensitivity analysis: Days on ventilation), and the whole sensitivity analysis can be viewed in the RMarkdown document containing the code.

Maternal education is excluded but SES is included in the models. The authors should explain how maternal SES was measured and classified.

Author’s response: Reviewer 1 Comment 3

In response to the reviewer’s helpful comment, we have now added the following sentence to our Methods:

“Maternal socioeconomic status (SES) values were extracted from the Carstairs index, which takes into consideration variables such as unemployment, car ownership, household overcrowding, and social class (Carstairs, 1991).”

Reviewer #2: PONE-D-20-37926

This study examines the associations between maternal stress and brain volumes in a group of 221 premature-born infants scanned around term age. They find weak associations between stressful life events and hippocampal volume, but these do not survive multiple comparison correction. An exploratory tensor-based morphometry analysis of the whole brain showed similar findings, suggesting no meaningful relationship between maternal stress and brain volumes in premature infants. Strengths of this study include the large sample and the two types of analysis to help confirm findings. The manuscript is well-written, and I think this study provides a valuable contribution to the literature to help understand the relationships between maternal stress and infant/child brain outcomes. I do have a few (mostly minor) suggestions to improve the manuscript.

1. The title of the manuscript refers to “structural brain development”, and this language appears elsewhere in the paper (e.g., p. 17 “associated with changes in brain volume”). However, this was a cross-sectional study that examined brain volumes at a single time point and did not measure changes over time, so I’d suggest the authors refer instead to “brain structure” or “brain volume” rather than brain development.

Author’s response: Reviewer 2 Comment 1

We appreciate this helpful comment and agree that using terms such as “development” and “changes” can be misleading to the reader. In response to this comment, we have made changes as outlined below:

We have changed the title of our manuscript from “Exploring the relationship between maternal prenatal stress and structural brain development in premature neonates” to “Exploring the relationship between maternal prenatal stress and brain structure in premature neonates”.

We have changed all instances in which we referred to “changes” or “development” in the context of cross-sectional research. Please see the “track changes” version of the manuscript and S1 Supplement to view the changes.

2. The stress and anxiety measures were administered after the child’s birth. While the authors do point to the stability of these reports over time, more information would be useful. Can you provide some citations for the stability of trait anxiety during pregnancy and the postpartum period? Having a preterm baby would be quite stressful; does this influence retrospective reports? It would be valuable to have more information about the stressful life events measure. Was the time period of reporting restricted to pregnancy, or did it include the time before pregnancy? What is the full range of possible scores for this stressful life events measure? Finally, it would be useful to briefly describe what you mean by “maternal anxiety/stress” in the abstract.

Author’s response: Reviewer 2 Comment 2

We thank the reviewer for this thoughtful comment, and for the opportunity to further clarify and discuss these issues. We are addressing each of the reviewer’s questions in turn, below.

“While the authors do point to the stability of these reports over time, more information would be useful. Can you provide some citations for the stability of trait anxiety during pregnancy and the postpartum period?”

We would like to draw the reviewer’s attention to S1 Supplement – Supporting Information, where we have included a section on “Additional information on anxiety and stressful life events”. In this section, we argue that trait anxiety is considered to be a relatively stable personality trait, while state anxiety reflects a transient anxious state. Here, we reference a study by Grant et al (2008) where self-reported trait anxiety is suggested to be relatively stable between pregnancy and 7 months postpartum. We also point the reader to several studies suggesting high reliability between data collected during pregnancy and self-report measures collected up to 30 years after birth (Tomeo et al., 1999, Quigley et al., 2007).

Having a preterm baby would be quite stressful; does this influence retrospective reports?

We agree with the reviewer that having a preterm baby is a stressful experience which could influence retrospective reports. This is the reason for our choice to use trait anxiety, over state anxiety measures. In response to the reviewer’s comments, we have added the following paragraph to S1 Supplement:

“Our decision to study trait anxiety instead of state anxiety is motivated by the fact that trait anxiety is considered to be a relatively stable personality trait, while state anxiety reflects a transient anxious state. State anxiety scores were not included in the analysis, as this measure was designed to be sensitive to the conditions under which the questionnaire is administered (Spielberger, 1972). It is likely that state anxiety scores would have been influenced by the stress-inducing situations of having a preterm baby, being in a hospital environment, and attending an appointment for an infant MRI scan, and thus would not be representative of prenatal anxiety levels.

In contrast, trait anxiety scores imply a generalized and enduring predisposition to respond to situations in an anxious manner, are less likely to be influenced by situational variables (Spielberger et al., 1972) and are thus more likely to be reflective of the mother’s prenatal anxiety levels. In the perinatal period, studies have reported that self-reported trait anxiety is not a transient state, but is relatively stable between pregnancy and 7 months postpartum (Grant et al., 2008)”

We would also like to draw the reviewer’s attention to our Methods section, where we have included the sentence “The analysis was restricted to trait anxiety, as it measures a relatively stable tendency to be prone to experiencing anxiety and thus extends to the period before birth “. To further emphasize this point, we have now also added the following to the Discussion section of the manuscript: “self-reported trait anxiety scores are relatively stable in the perinatal period (Grant et al., 2008) (See S1 Supplement for further discussion).”

Lastly, to further clarify, we have also added the following information to S1 Supplement:

“It is important to acknowledge the possibility that focusing on trait anxiety rather than state anxiety could have influenced our findings. While some previous studies in infants have reported changes in brain development related to maternal state anxiety (Dean et al., 2018) or a combined score of state and trait anxiety (Rifkin-Graboi et al., 2015), these studies analysed white matter microstructure using diffusion MRI. To our knowledge, the only other study which has used the STAI to assess changes in brain volume in infants (Qiu et al., 2013), only included trait anxiety measures. Their results were in line with our current findings, as they reported that maternal trait anxiety was not associated with any differences in hippocampal volume at birth.”

It would be valuable to have more information about the stressful life events measure. Was the time period of reporting restricted to pregnancy, or did it include the time before pregnancy? What is the full range of possible scores for this stressful life events measure?

To answer the question regarding the time period of reporting, we would like to draw the reviewer’s attention to the following sentence in the manuscript’s Methods section “which included yes/no answers to a list of potentially stressful life events the participant may have experienced in the year prior to the study visit”. This period includes the pregnancy and several months beforehand.

We agree with the reviewer that further information would be helpful. In response to this comment, we have now added an additional section in S1 Supplement, named “Additional information regarding the stressful life events measure”. This details the list of events and the frequency of responses per each item, as well as additional information regarding how the scores were computed.

Finally, it would be useful to briefly describe what you mean by “maternal anxiety/stress” in the abstract.

We have now clarified this in the abstract, by specifying “trait anxiety and stressful life events”

3. Why was the study-specific template for analysis based on 161 infants instead of the full sample? Note that I’m not suggesting you need to redo this, but some rationale would be helpful.

Author’s response: Reviewer 2 Comment 3

We thank the reviewer for the opportunity to clarify this analytical decision. The reason to use a sub-sample of the population was primarily to reduce computational load. Please note that increasing sample size has been reported to have minimal impact in template creation after a certain threshold (Yang et al., 2020).

In response to this comment, we have now clarified this in the manuscript (please see Methods and Materials – Tensor Based Morphometry – Template construction) by adding the following sentence:

“In order to reduce computational load, a smaller subset of 161 images meeting inclusion criteria (i.e. PMA at scan <45 weeks, no major lesions, and of good quality) were used to build the population template for this study.”

4. Women who disclosed alcohol and/or drug abuse were excluded. Were women excluded for any use at all? (in which case, should say “use” instead of “abuse”) If not, please define what you mean by alcohol/drug abuse, and what the cutoffs were.

Author’s response: Reviewer 2 Comment 4

This variable was based on self-disclosure by the mother and thus no further information is available regarding cutoffs. No further information was collected regarding alcohol and drug use.

5. Please define units in Table 1 (e.g., for maternal education, birthweight, GA, PMA, OFC).

Author’s response: Reviewer 2 Comment 5

In response to the reviewer’s comment, we have defined the units in Table 1 as follows: maternal education (years), birth weight (grams), GA at birth (weeks), PMA at scan (weeks), OFC at birth (cm).

6. Preterm infants have brain differences from full-term infants, and it is possible that associations between maternal stress/anxiety and brain volumes are masked by differences caused by preterm birth. Therefore, while this study represents an important contribution to the literature, the findings cannot necessarily be generalized to full-term born children. I think the differences between preterm infant brains and term-born infant brains, and the implications for these findings merits some discussion.

Author’s response: Reviewer 2 Comment 6

We thank the reviewer for these insightful comments. The aim of our study was to address a gap in literature, as studies reporting associations between maternal prenatal stress and early brain development have focused specifically on term-born infants. We believe that it is important to understand whether these findings are also observed in preterm-born children.

In response to the reviewer’s comment, we have added a paragraph to the Discussion, calling for future research to include term-born controls:

“It is important to highlight that our sample consisted of preterm infants, a population known to have regional brain volume abnormalities (Peterson et al., 2000) and adverse neuropsychiatric and developmental outcomes (Allen et al., 2008, Nosarti et al., 2012). We caution against generalizing these findings to infants born at term, and suggest that further studies with term-born controls are needed to further clarify the role that early adverse experiences such as maternal stress may have in moderating the association between preterm birth and adverse outcomes in this vulnerable population. “

In addition, we expanded on this argument in S1 Supplement, by adding the following paragraph in the “Prematurity and brain development section”:

“Preterm infants are known to be at risk for adverse neuropsychiatric and developmental outcomes (e.g. Allen et al., 2008, Nosarti et al., 2012). A large number of research studies have focused on investigating the relationship between brain development and these adverse outcomes (Ment et al., 2009, Counsell et al., 2008, Peterson et al., 2000). However, it is essential to also assess the role that early adverse experiences may have in moderating these associations. In a recent study by Benavente-Fernandez et al (2019), the association between brain injury and cognitive outcomes in a sample of children born preterm (24-32 weeks GA) was mediated by maternal socioeconomic status. Similarly, it is possible that exposure to maternal prenatal stress may exacerbate the risk for negative outcomes in preterm-born children. Our findings in Lautarescu et al. (2020) suggested that maternal prenatal stress is related to alterations in white matter microstructure, and thus provided evidence that maternal stress may impact early brain development not only in term-born children, but also in children born preterm. We believe that the present study represents an important contribution to the literature, by also focusing on brain volumes in an overlapping sample of preterm infants. Further research including term-born controls is required to further clarify the nature of this relationship, in order to develop potential interventions that may dampen or reverse the effects of early adversity.”

7. It is disappointing that the data is not publicly available. Ethics constraints may restrict data sharing, but directing inquiries to your local ethics board is not helpful. Perhaps interested parties could contact the authors instead?

Author’s response: Reviewer 2 Comment 7

We have now shared a minimal de-identified data set (S3. Dataset). Please see the section “Additional information regarding data and code” in “S1 Supplement” for details regarding this dataset.

We have now also provided contact details for our local ethics board. We have not provided contact details for the authors for data enquiries, as PLOS ONE Data Availability Policy states that “It is not acceptable for an author to be the sole named individual responsible for ensuring data access”.

We have now updated our data availability statement as follows:

“Relevant data have been shared within the Supporting Information files. This data includes the majority of the variables included in the regression models reported in the manuscript. As this data contained sensitive and potentially identifiable information, we were unable to share the full anonymised dataset necessary to replicate the study findings. Data for the variable "maternal age" could not be shared, as this combination of variables would increase the risk of the data being identifiable. Requests related to data access can be directed to the Hammersmith and Queen Charlotte’s Research Ethics Committee ([westlondon.rec@hra.nhs.uk)](mailto:westlondon.rec@hra.nhs.uk)).”

8. Typo/word errors:
a. In the methods, “we inputted missing values”; do you mean imputed?
b. P. 9, line 202 should likely say “constraints” instead of “restraints”
c. P. 4, line 98 “women disclosed alcohol” needs “who” inserted

Author’s response: Reviewer 2 Comment 8

We thank the reviewer for pointing out these typos. We have now corrected the mistakes pointed out in 8a. and 8b.

Regarding comment 8c, the full sentence reads “We excluded cases where (...) women disclosed alcohol and/or drug abuse “, and thus does not need a “who”.

6. PLOS authors have the option to publish the peer review history of their article ([what does this mean?](https://eur03.safelinks.protection.outlook.com/?url=https%3A%2F%2Fjournals.plos.org%2Fplosone%2Fs%2Feditorial-and-peer-review-process%23loc-peer-review-history&data=04%7C01%7Calexandra.lautarescu%40kcl.ac.uk%7C5a1f561de88944cf265808d8c24b5483%7C8370cf1416f34c16b83c724071654356%7C0%7C0%7C637472976113192814%7CUnknown%7CTWFpbGZsb3d8eyJWIjoiMC4wLjAwMDAiLCJQIjoiV2luMzIiLCJBTiI6Ik1haWwiLCJXVCI6Mn0%3D%7C1000&sdata=hcWBy8XfVIfZsnSwhdjsGkYe2mn6PkdG%2FhVYe852JUc%3D&reserved=0)). If published, this will include your full peer review and any attached files.

If you choose “no”, your identity will remain anonymous but your review may still be made public.

**Do you want your identity to be public for this peer review?** For information about this choice, including consent withdrawal, please see our [Privacy Policy](https://eur03.safelinks.protection.outlook.com/?url=https%3A%2F%2Fwww.plos.org%2Fprivacy-policy&data=04%7C01%7Calexandra.lautarescu%40kcl.ac.uk%7C5a1f561de88944cf265808d8c24b5483%7C8370cf1416f34c16b83c724071654356%7C0%7C0%7C637472976113202810%7CUnknown%7CTWFpbGZsb3d8eyJWIjoiMC4wLjAwMDAiLCJQIjoiV2luMzIiLCJBTiI6Ik1haWwiLCJXVCI6Mn0%3D%7C1000&sdata=Dm1Eihh1tb1kEhDwnm9waFQKECfuzs7E0HvJ5Pn9uPs%3D&reserved=0).

Reviewer #1: No

Reviewer #2: No

References

Allen, M. C. (2008). Neurodevelopmental outcomes of preterm infants. *Current opinion in neurology*, 21(2), 123-128.

Benavente-Fernández, I., Synnes, A., Grunau, R. E., Chau, V., Ramraj, C., Glass, T., ... & Miller, S. P. (2019). Association of socioeconomic status and brain injury with neurodevelopmental outcomes of very preterm children. *JAMA network open, 2*(5), e192914-e192914.

Carstairs V, Morris R. (1991): *Deprivation and Health in Scotland*. Aberdeen University Press

Counsell SJ, Edwards AD, Chew AT, Anjari M, Dyet LE, Srinivasan, L. et al. (2008). Specific relations between neurodevelopmental abilities and white matter microstructure in children born preterm. *Brain, 131*(12), 3201-3208.

Dean DC, Planalp EM, Wooten W, Kecskemeti SR, Adluru N, Schmidt CK et al. (2018): Association of prenatal maternal depression and anxiety symptoms with infant white matter microstructure. *JAMA pediatrics, 172*(10), 973-981.

Edwards, A. D., Redshaw, M. E., Kennea, N., Rivero-Arias, O., Gonzales-Cinca, N., Nongena, P., ... & Counsell, S. (2018). Effect of MRI on preterm infants and their families: a randomised trial with nested diagnostic and economic evaluation. *Archives of Disease in Childhood-Fetal and Neonatal Edition, 103*(1), F15-F21.

Grant, K. A., McMahon, C., & Austin, M. P. (2008). Maternal anxiety during the transition to parenthood: a prospective study. *Journal of affective disorders, 108(*1-2), 101-111.

Lautarescu, A., Pecheva, D., Nosarti, C., Nihouarn, J., Zhang, H., Victor, S., ... & Counsell, S. J. (2020). Maternal prenatal stress is associated with altered uncinate fasciculus microstructure in premature neonates. *Biological psychiatry, 87*(6), 559-569.

Ment, L. R., Hirtz, D., & Hüppi, P. S. (2009). Imaging biomarkers of outcome in the developing preterm brain. *The Lancet Neurology, 8*(11), 1042-1055.

Nosarti C, Reichenberg A, Murray RM, Cnattingius S, Lambe MP, Yin L et al. (2012). Preterm birth and psychiatric disorders in young adult life. *Archives of general psychiatry, 69*(6), 610-617.

Peterson, B. S., Vohr, B., Staib, L. H., Cannistraci, C. J., Dolberg, A., Schneider, K. C., ... & Duncan, C. C. (2000). Regional brain volume abnormalities and long-term cognitive outcome in preterm infants. *Jama, 284*(15), 1939-1947.

Qiu, A., Rifkin-Graboi, A., Chen, H., Chong, Y. S., Kwek, K., Gluckman, P. D., ... & Meaney, M. J. (2013). Maternal anxiety and infants' hippocampal development: timing matters*. Translational psychiatry, 3*(9), e306-e306.

Quigley, M. A., Hockley, C., & Davidson, L. L. (2007). Agreement between hospital records and maternal recall of mode of delivery: evidence from 12 391 deliveries in the UK Millennium Cohort Study*. BJOG: An International Journal of Obstetrics & Gynaecology, 114*(2), 195-200.

Rifkin-Graboi A, Meaney MJ, Chen H, Bai J, Bak W, Thway Tint M et al. (2015): Antenatal Maternal Anxiety Predicts Variations in Neural Structures Implicated in Anxiety Disorders in Newborns. *Journal of the American Academy of Child & Adolescent Psychiatry, 54*(4), 313–321

Spielberger, C. D. (1972). Anxiety as an emotional state. *Anxiety-Current trends and theory, 3*-20.

Tomeo, C. A., Rich-Edwards, J. W., Michels, K. B., Berkey, C. S., Hunter, D. J., Frazier, A. L., ... & Buka, S. L. (1999). Reproducibility and validity of maternal recall of pregnancy-related events. *Epidemiology, 7*74-777.

Yang, G., Zhou, S., Bozek, J., Dong, H. M., Han, M., Zuo, X. N., ... & Gao, J. H. (2020). Sample sizes and population differences in brain template construction. *NeuroImage, 206,* 116318.
